# Oncogene or tumor suppressor gene: An integrated pan-cancer analysis of *NBPF1*

**DOI:** 10.3389/fendo.2022.950326

**Published:** 2022-08-17

**Authors:** Lei Li, Sen Chen, Yueming Tang, Jie Wu, Yangzhige He, Ling Qiu

**Affiliations:** ^1^ Department of Laboratory Medicine, Peking Union Medical College Hospital, Peking Union Medical College and Chinese Academy of Medical Science, Beijing, China; ^2^ Immune Cells and Antibody Engineering Research Center of Guizhou Province, Key Laboratory of Biology and Medical Engineering, School of Biology and Engineering, Guizhou Medical University, Guiyang, China; ^3^ Department of Medical Research Center, State Key Laboratory of Complex Severe and Rare Diseases, Peking Union Medical College Hospital, Chinese Academy of Medical Science and Peking Union Medical College, Beijing, China; ^4^ State Key Laboratory of Complex Severe and Rare Diseases, Peking Union Medical College Hospital, Peking Union Medical College and Chinese Academy of Medical Science, Beijing, China

**Keywords:** *NBPF1*, pan-cancer analysis, adrenocortical carcinoma, immune, biomarker

## Abstract

Neuroblastoma breakpoint family, member 1 (*NBPF1*), appears to be a double-edged sword with regard to its role in carcinogenesis. On the one hand, the tumor-suppressing functions of *NBPF1* have been definitively observed in neuroblastoma, prostate cancer, cutaneous squamous cell carcinoma, and cervical cancer. On the other hand, there is evidence that *NBPF1* regulates the colony formation, invasion, and maintenance of liver cancer cells and hence functions as an oncogene. The roles of *NBPF1* are strictly dependent on the biological context and type of organization. However, a systematic pan-cancer analysis has thus far not been undertaken, and the significance of *NBPF1* in the occurrence and progression of many malignancies is uncertain. In this paper, bioinformatics techniques were employed to analyze *NBPF1* expression across different cancers and investigate the relationship between *NBPF1* and clinical features, prognosis, genetic alteration, and tumor immune microenvironment, respectively. Our results show that *NBPF1* is variably expressed in distinct tumor tissues and is also closely linked to clinical outcomes. In particular, compared to other tumor types, there was a strong negative correlation between *NBPF1* expression and various components of the tumor microenvironment in adrenocortical carcinoma (ACC). We thus developed an *NBPF1*-derived immune risk model based on *NBPF1*-related immune genes; ACC patients with a high-risk score tended to have a poorer prognosis, accompanied by immune hyporesponsiveness. *NBPF1* can be used as a prognostic biomarker for multiple cancers. Moreover, anti-*NBPF1* immunotherapy may be suitable for treating ACC patients.

## Introduction

The increasing sophistication of sequencing technologies, as well as bioinformatics, has allowed us to identify tumor driver genes, molecular subtypes, and other features using large-scale cancer genomics programs, such as The Cancer Genome Atlas (TCGA), the International Cancer Genome Consortium (ICGC), and GENIE. These programs have provided large amounts of tumor genomic and clinical data resources. The structured integration of various multi-omics platforms provides an important opportunity to understand the major genetic alterations that drive cancer development and progression. As genomic and molecular interactions are more reliably described, it is becoming increasingly clear that understanding the characteristics of a unique tissue environment, altered pathways, and immunological features of each cancer and subtype are essential to discern the underlying dynamics of tumorigenesis and to inform diagnosis, prognosis, and treatment.

The neuroblastoma breakpoint family, member 1 (*NBFP1*) gene was first reported to be disrupted by constitutional translocation in a neuroblastoma patient (1, 2). Subsequently, numerous studies have reported that chromosomal region 1p36, where *NBPF1* is located, is broadly deleted in various human malignancies, such as those of hematopoietic, epithelial, and neural origins (3). A meta-analysis based on the Oncomine database showed that levels of *NBPF1* were decreased in neuroblastomas that recurred within five years (4). Additionally, in cutaneous squamous cell carcinoma and cervical cancer, *NBPF1* inhibits tumor cell proliferation, growth, and cell cycle progression by targeting the PI3K/mTOR and Akt-p53-Cyclin signaling pathways, respectively (5, 6). However, Wu et al. reported that downregulation of *NBPF1* in human liver cancer cells strongly inhibits their proliferative capacity (7). Moreover, *NBPF1* can form a trimolecular complex with chibby and clusin to engage in critical signaling pathways (such as the Wnt-catenin and NF-kappa-B signaling pathways), which are activated in a disease type-specific manner (8–11). Collectively, previous research has shown that *NBPF1* has diverse functions in carcinogenesis and clinical prognosis. However, the expression and prognostic implications of *NBPF1* are still unknown, and its probable role in pathological and physiological activities is yet to be thoroughly studied.

## Materials and methods

### Gene expression

The “Gene_DE” function of the Tumor Immune Estimation Resource, version 2 tool (TIMER2, http://timer.cistrome.org/) was used for observing the *NBPF1* transcription differences between 33 tumors and matched noncancerous tissues, including: adrenocortical carcinoma (ACC) (n = 79), bladder urothelial carcinoma (BLCA) (n = 408), breast invasive carcinoma (BRCA) (n = 1,093), cervical squamous cell carcinoma and endocervical adenocarcinoma (CESC) (n = 304), cholangiocarcinoma (CHOL) (n = 36), colon adenocarcinoma (COAD) (n = 457), lymphoid neoplasm diffuse large B cell lymphoma (DLBC) (n = 48), esophageal carcinoma (ESCA) (n = 184), glioblastoma multiforme (GBM) (n = 153), head and neck squamous cell carcinoma (HNSC) (n = 520), kidney chromophobe renal cell carcinoma (KICH) (n = 66), kidney renal clear cell carcinoma (KIRC) (n = 533), kidney renal papillary cell carcinoma (KIRP) (n = 290), acute myeloid leukemia (LAML) (n = 173), brain lower grade glioma (LGG) (n = 516), liver hepatocellular carcinoma (LIHC) (n = 371), lung adenocarcinoma (LUAD) (n = 515), lung squamous cell carcinoma (LUSC) (n = 501), mesothelioma (MESO) (n = 87), ovarian serous cystadenocarcinoma (OV) (n = 303), pancreatic adenocarcinoma (PAAD) (n = 178), pheochromocytoma and paraganglioma (PCPG) (n = 179), prostate adenocarcinoma (PRAD) (n = 497), rectum adenocarcinoma (READ) (n = 166), sarcoma (SARC) (n = 259), skin cutaneous melanoma (SKCM) (n = 103), stomach adenocarcinoma (STAD) (n = 415), testicular germ cell tumors (TGCT) (n = 150), thyroid carcinoma (THCA) (n = 501), thymoma (THYM) (n = 120), uterine corpus endometrial carcinoma (UCEC) (n = 545), uterine carcinosarcoma (UCS) (n = 57), and uveal melanoma (UVM) (n = 80).

Gene expression data were formatted in transcripts per million reads (TPM), and relevant patient records of 33 cancer types were extracted from the TCGA database (http://cancergenome.nih.gov/). Clinical data from the TCGA dataset are shown in **Supplementary Tables 1, 2**. Microarray datasets with detailed survival data of the ACC cohort, namely, GSE33371 and GSE10927, were acquired from the GEO database (https://www.ncbi.nlm.nih.gov/geo/) and were set as external validation datasets. A total of 47 ACC specimens with accessible prognostic information were included in the external validation dataset.

The limma R package was used to investigate the correlation between clinicopathological features, including pathological stage, TNM stage, and *NBPF1* levels.

The TISIDB online tool (http://cis.hku.hk/TISIDB/) was used to analyze *NBPF1* expression across different tumor subtypes.

### Immunohistochemistry staining

The Human Protein Atlas (HPA; http://www.proteinatlas.org/) was used to obtain IHC images of *NBPF1* protein expression.

### Survival prognosis analysis of *NBPF1*

The Gene Expression Profiling Interactive Analysis, version 2 (GEPIA2, https://gepia2.cancer-pku.cn/#analysis) webserver was used to extract survival data relating to *NBPF1*, including Overall Survival (OS) and Disease-Free Survival (DFS). The median value of *NBPF1* expression was set as the expression threshold to determine the high or low expression of *NBPF1*.

A univariate Cox regression analysis was used to analyze the association between levels of *NBPF1* and survival parameters, including OS and disease-specific survival (DSS) in 33 cancers.

Moreover, to determine whether *NBPF1* expression could independently predict OS or DSS in patients with ACC, the survival R package was applied to undertake univariate and multivariate logistic regression analysis with *NBPF1* expression and various clinical features as variables.

### Genetic alteration analysis

The cBioPortal online tool (https://www.cbioportal.org/) was used to assess the frequency of *NBPF1* genetic changes among the TCGA tumors. We further analyzed the correlation between survival metrics and *NBPF1* DNA copy number amplification and methyltransferase using the GSCA database (http://bioinfo.life.hust.edu.cn/GSCA/).

### Correlation between *NBPF1* expression and immunological characteristics

The TISIDB online tool was used to assess *NBPF1* expression across different immune subtypes and to quantify the linkages between *NBPF1* expression and tumor-infiltrating lymphocyte (TIL) abundance. Moreover, we used the TIMER2 web browser to retrieve immune infiltration data for all 33 cancer types. Eight different algorithms, namely, CIBERSORT, CIBERSORT-ABS, EPIC, MCP-counter, quantTIseq, xCell, TIMER, and TIP, were used to estimate immune infiltration.

We downloaded the immune cell infiltration data and immune process score data from the TIP database (12). The gsva R package was employed to conduct ssGSEA analysis for evaluating tumor infiltration fraction, with the generated heatmap showing the correlation between *NBPF1* expression levels and the abundance of immune infiltrate cells. The ESTIMATE R package was used to evaluate the stromal and immune scores. Moreover, 122 immunomodulators, including MHC, receptors, chemokines, and immune stimulators, and 47 common immune checkpoint genes were selected from the previously published studies by Charoentong et al. and Chen et al. (13, 14). The Spearman’s correlations between *NBPF1* expression levels and immunomodulators and immune checkpoints were calculated in R. PERL scripts were used to evaluate the tumor mutational burden (TMB) of each TCGA tumor case based on the somatic mutation data obtained from the TCGA. The tumor mutation load was calculated as the total number of mutations per megabase sequence in the exon-coding region of a gene (15).


$$\begin{array}{l} TMB\;(mut/mb)=total\;number\;of\;mutations\;(including\;synonymous,\;nonsynonymous\;point\;mutaion,\;\\ substitutions,\;insertions,\;and\;deletions)/size\;of\;the\;coding\;region\;of\;the\;target\;region \end{array}$$

### Identification of differentially expressed genes and functional enrichment analysis

The limma R package was used to identify the differentially expressed genes (DEGs) with a |log2(FC)| >1, and the statistical significance of the adjusted P-value was set at<0.05.

To learn more about the probable functions of DEGs, we used the clusterProfiler R package for Gene Ontology (GO)/Kyoto Encyclopedia of Genes and Genomes (KEGG) and GSEA enrichment analysis, the org.Hs.eg.db R package for ID reformation, and the GOplot R package for the z-score calculation. The Zscore was used to determine whether the corresponding entry was positively regulated (zscore >0) or negatively regulated (zscore<0). The generated circle map shows the data of the top five GO terms (including biological process, cellular component, and molecular function) and KEGG functional enrichment of DEGs.

Furthermore, the GSEA R package was used to evaluate the correlation between the marker gene and all other genes contained in the external validation datasets. All genes were then ranked from high to low according to their correlations, and this sorted gene set was used in our analysis. The KEGG signaling pathway set was used as a preset set for the identification of the clusters of biologically related enriched meta-pathways of *NBPF1*. Statistical significance was set at P<0.05. The enrichment results are presented in **Supplementary Table 3**.

### Establishment of an immune risk score

Evaluation of the Import Shared Database (https://www.immport.org/shared/home) yielded 1,811 immune-related genes (IRGs). The shared immune-related differentially expressed genes (IDEGs) between DEGs and IRGs were identified using the Venn Diagram R program.

We further conducted a univariate Cox regression analysis to screen out the immune-related prognostic genes (IRPGs) with the ability to affect ACC prognosis *via* the survival R package. Statistical significance was set at P<0.05. The TCGA-ACC cohort was separated into training and validation sets at a 1:1 ratio. Based on the training cohort, LASSO regression analysis was conducted to screen out the optimal candidate IRPGS and establish an immune risk signature in these IRPGS *via* the glmnet package. According to the prognostic model, the risk score of each sample in the training cohort was calculated, and the median risk score was set as the cut-off value to divide all ACC patients into high- and low-risk groups. We evaluated the effect of the immune risk signature on ACC prognosis between the high- and low-risk groups in the training, testing, and validation cohorts using Kaplan–Meier curves. Subsequently, ROC curves and principal component (PCA) analysis were applied to evaluate the prediction accuracy.

After univariate and multivariate Cox analyses, the overall survival rates of each patient at 1, 2, and 3 years were predicted using a nomogram that integrated ACC clinical features and immune risk signatures. The corresponding calibration curve was used to estimate the prediction accuracy of the nomogram. The heatmap package was used to draw a heatmap of the clinical features and IRS RNA expression.

Since the IRS was built based on IDEGs, we evaluated the correlation of the immune risk signature with immunological characteristics among high- and low-risk populations in the TCGA-ACC cohort. First, the CIBERSORT algorithm and LM22 feature matrix were applied to calculate the composition of immune cells between the two risk groups. Second, the ESTIMATE R package, which generates an ESTIMATEScore inferring tumor purity, was used to evaluate the difference in ESTIMATEScore between high- and low-risk ACC patients. Third, the TIDE algorithm helped us separate ACC patients into non-responder and responder groups according to their clinical response to immunotherapy. Statistical significance was set at P<0.05. Fourth, the expressive variability of immune checkpoints among high- and low-risk populations was determined.

### Assessment of drug sensitivity

The Corrplot R package was used to measure the correlations between IRPG expression levels and drug sensitivity using the NCI-60 analysis tools in CellMiner (https://discover.nci.nih.gov/cellminer/). Statistical significance was set at P<0.05. The scatter diagrams generated showed significant correlations with a Pearson correlation coefficient (Cor) >0.6 and a p-value<0.05.

### Statistical analysis

The Kruskal–Wallis (KW) test, one-way ANOVA, and Wilcoxon rank sum test were used to analyze *NBPF1* expression among 33 TCGA cancers with distinct pathological phases and TNM. Then, Spearman’s method was applied to calculate the correlation between two variables, and statistical significance was set at P<0.05. The R version used was 3.6.3.

## Results

### Analysis of *NBPF1* expression across multiple cancer types

**Figure 1:**
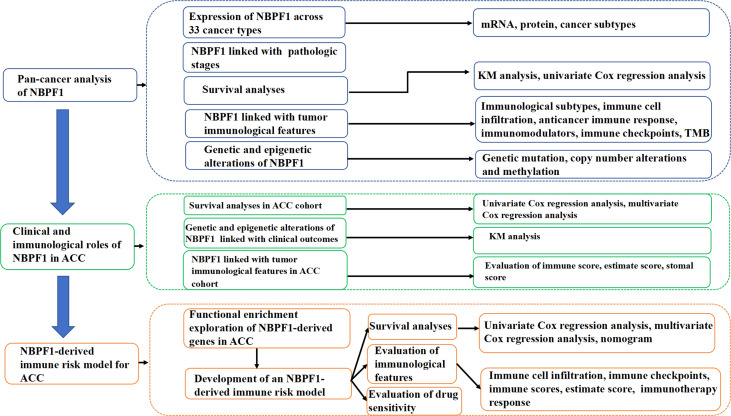
The analysis and indicators involved in the study.

**Figure 2:**
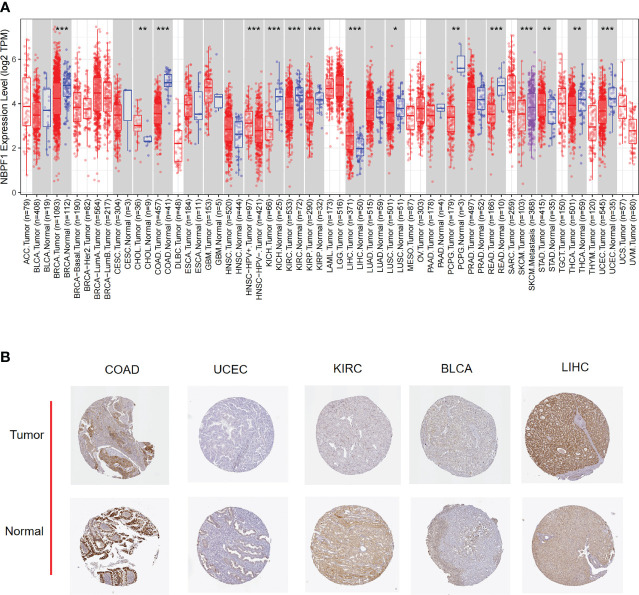
The expression of *NBPF1* in human cancers. **(A)** The expression status of the *NBPF1* gene in normal tissues and tumors taken from the TIMER2 database. **(B)** *NBPF1* protein expression based on immunohistochemistry staining taken from the Human Protein Atlas database. (*P<0.05; **P < 0.01; ***P < 0.001).

Details of this analysis are shown in **Figure 1**. First, we used the TIMER database to ascertain the mRNA expression of *NBPF1* in multiple cancers and corresponding noncancerous tissues, which revealed that *NBPF1* was differentially expressed in various cancers. As shown in **Figure 2A**, *NBPF1* tended to be significantly downregulated in most cancer types, including BRCA, COAD, KICH, KIRC, KIRP, LUSC, PCPG, READ, THCA, and UCEC, suggesting that *NBPF1* has an essential anti-cancer role in these cancers, whereas it is overexpressed in CHOL, LIHC, and STAD. In addition to transcription, we investigated the protein expression of *NBPF1* in the HPA database since only 12 types of cancer, namely, cervical, colorectal, endometrial, liver, lung, pancreatic, prostate, renal, skin, stomach, testis, and thyroid cancers, were available with normal tissues as controls. We found that, compared with the corresponding normal tissue, *NBPF1* was differentially expressed in colorectal, endometrial, liver, renal, stomach, and thyroid cancers (**Figure 2B**). However, the expression in the other six tumors did not differ from that in normal tissues.

**Figure 3:**
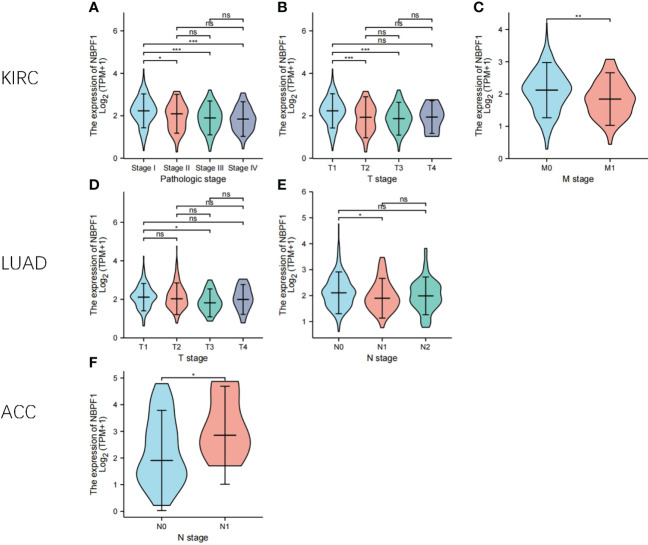
The expression levels of *NBPF1* in tumors with different pathological stages. **(A–F)** The pathological stage-dependent expression levels of *NBPF1* (*P < 0.05; **P < 0.01; ***P < 0.001) ns, no significant difference.

Next, we examined the expression profile of *NBPF1* in tumors of various pathological stages and cancer subtypes, which indicated that low expression of *NBPF1* generally predicted late pathological stage in KIRC and LUAD, larger tumor size in KIRC and LUAD, more lymph node metastases in KIRC, and more distant metastases in LUAD. However, an exception to the rule was found in ACC, where overexpression of *NBPF1* predicted a greater possibility of lymph node involvement (**Figures 3A–F**). Additionally, there were discrepancies in the expression levels of *NBPF1* in diverse cancer subtypes (**Supplementary Figure 1**). For example, *NBPF1* expression was found to be higher in the CIMP-high ACC subtype.

### Analysis of the prognostic significance of *NBPF1* across multiple cancer types

**Figure 4:**
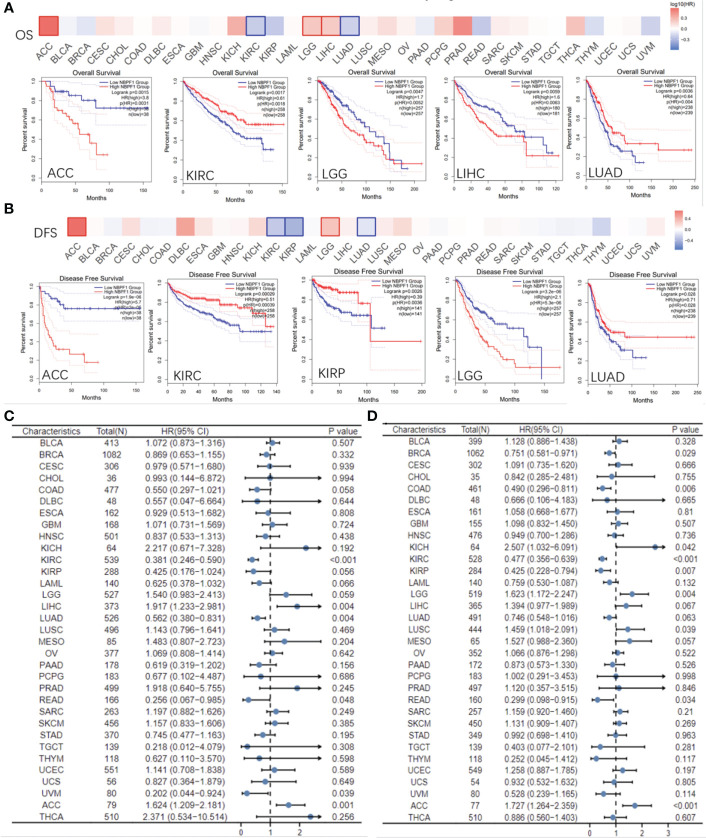
Prognostic value of *NBPF1* in human cancers. KM curves and heatmap showing the relationship between *NBPF1* gene expression levels and **(A)** overall survival and **(B)** disease-free survival taken from the GEPIA2 database. Forest plots of pan-cancer univariate Cox regression analyses of **(C)** overall survival and **(D)** disease-specific survival.

Based on the typical expression values of *NBPF1* in 33 types of cancer, patients were separated by median into *NBPF1*-high and *NBPF1*-low transcription groups. The correlation between *NBPF1* transcription and survival metrics, including OS and DFS, was estimated *via* Kaplan–Meier survival curves. In patients with ACC, LGG, and LIHC, increased *NBPF1* transcription was a risk factor for poorer OS and DFS, while increased *NBPF1* expression corresponded with better OS and DFS in patients with KIRC and LUAD (**Figures 4A, B**).

Reduced *NBPF1* mRNA expression was also associated with shorter OS in KIRC and LUAD according to univariate Cox regression analysis. In contrast, increased *NBPF1* expression was correlated with unfavorable OS in ACC and LIHC. We also noticed that *NBPF1* expression had a protective influence on DSS in BRCA, COAD, KICH, KIRC, and KIRP. On the other hand, *NBPF1* expression was found to be a risk factor for poor DSS in ACC, LGG, and LUSC patients (**Figures 4C, D**).

These results indicate that *NBPF1* is a reliable prognostic biomarker, especially for ACC. We then conducted univariate and multivariate Cox regression analyses to evaluate whether clinical parameters and the expression of *NBPF1* were independent prognostic factors for OS and DSS in ACC patients. **Tables 1**, **2** show that *NBPF1* was an appropriate independent prognostic tool for patients with ACC (OS: univariate: HR = 1.624, 95% CI = 1.209–2.181, P = 0.001; multivariate: HR = 1.431, 95% CI = 1.031–1.986, P = 0.032); (DSS: univariate: HR = 1.727, 95% CI = 1.264–2.359, P<0.001; multivariate: HR = 1.489, 95% CI = 1.060–2.091, P = 0.022).

**Table 1:** Univariate and multivariate Cox regression analyses of clinical characteristics associated with OS of ACC.

Characteristics	Total (N)	Univariate analysis		Multivariate analysis	
		Hazard ratio (95% CI)	P-value	Hazard ratio (95% CI)	P-value
T stage	77				
T1 and T2	51	Reference			
T3 and T4	26	10.286 (3.976–26.608)	**<0.001**	6.963 (0.892–54.331)	0.064
N stage	77				
N0	68	Reference			
N1	9	2.038 (0.769–5.400)	0.152		
M stage	77				
M0	62	Reference			
M1	15	6.150 (2.710–13.959)	**<0.001**	1.821 (0.707–4.693)	0.214
Pathologic stage	77				
Stage I and Stage II	46	Reference			
Stage III and Stage IV	31	6.476 (2.706–15.498)	**<0.001**	0.827 (0.099–6.939)	0.861
*NBPF1*	79	1.624 (1.209–2.181)	**0.001**	1.431 (1.031–1.986)	**0.032**

Significant results (P < 0.05) are bolded.

**Table 2:** Univariate and multivariate Cox regression analyses of clinical characteristics associated with DSS of ACC.

Characteristics	Total (N)	Univariate analysis		Multivariate analysis	
		Hazard ratio (95% CI)	P-value	Hazard ratio (95% CI)	P-value
T stage	76				
T1 and T2	51	Reference			
T3 and T4	25	9.927 (3.812–25.853)	<0.001	6.730 (0.860–52.685)	0.069
N stage	76				
N0	67	Reference			
N1	9	2.115 (0.795–5.630)	0.134		
M stage	76				
M0	62	Reference			
M1	14	5.880 (2.549–13.568)	<0.001	1.729 (0.660–4.526)	0.265
Pathologic stage	76				
Stage I and Stage II	46	Reference			
Stage III and Stage IV	30	6.256 (2.596–15.079)	<0.001	0.826 (0.099–6.926)	0.860
*NBPF1*	77	1.727 (1.264–2.359)	<0.001	1.489 (1.060–2.091)	0.022

### Analysis of genetic alteration in *NBPF1* across multiple cancer types

**Figure 5:**
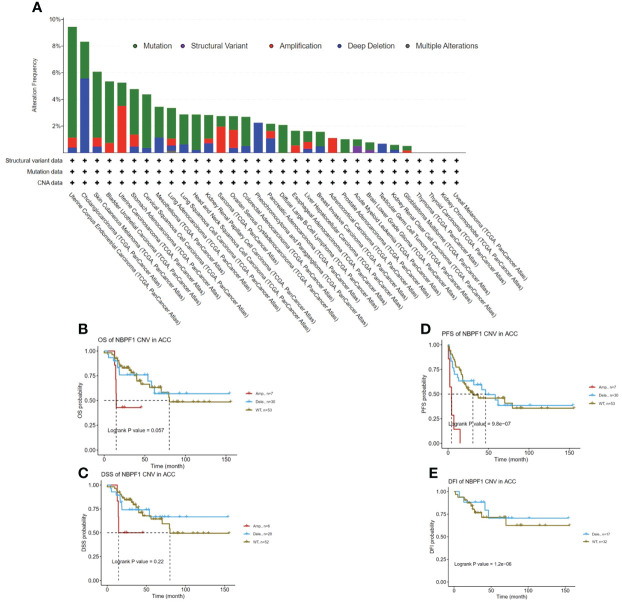
Genetic alteration of *NBPF1* in TCGA tumors. **(A)** Alteration frequency and mutation type of *NBPF1*. **(B–E)** Correlation between mutation status and OS, DSS, PFS, and DFI in ACC patients.

We observed the genetic alteration status of *NBPF1* in 33 types of cancer in the TCGA cohort. These consisted primarily of mutation status, copy number alterations (CNA), and methylation. Here, the mutation frequency was higher in UCEC and CHOL than in the other cancers. Additionally, the genomic alteration frequency of *NBPF1* was less than 2% in ACC, where the “amplification” type of CNA was the main alteration (**Figure 5A**). Copy number alterations of *NBPF1* were associated with the clinical survival prognosis of ACC patients (**Figure 5A–E**). In particular, for patients had copy number amplification of *NBPF1*, their disease-free interval (DFI) and progression-free survival (PFS) rate was worse than those without *NBPF1* alteration or those with copy number deletion alterations (**Figure 5D–E**). Furthermore, a strong negative correlation was found between *NBPF1* transcription and DNA methylation in 14 types of cancers (**Supplementary Figure 2**), and we also observed that patients with ACC with higher DNA methylation of *NBPF1* showed a better prognosis in PFS. According to the above data, *NBPF1* may be highly expressed in the ACC by underlying DNA copy number amplification or methylation variants, resulting in worse survival (**Figures 5B, C**).

### The immunological role of *NBPF1* across multiple cancer types

**Figure 6:**
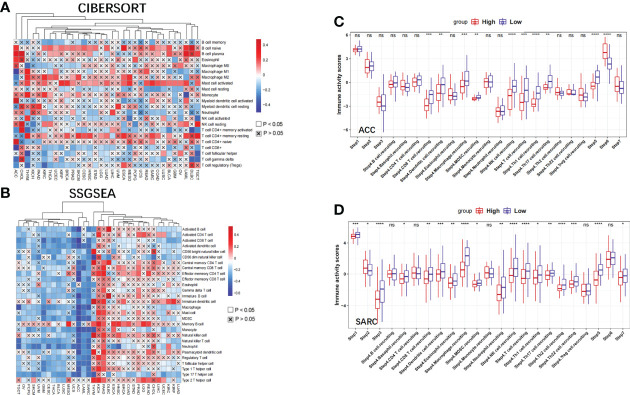
The effect of *NBPF1* on immunological status in human cancers. **(A)** Correlation analysis between *NBPF1* expression and the abundance of tumor-infiltrating lymphocytes in human cancers using the CIBERSORT algorithm. **(B)** Correlation between *NBPF1* and 28 tumor-associated immune cells using the ssGSEA algorithm. **(C, D)** Comparison between groups with high and low *NBPF1* expression in the various stages of the cancer immune cycle in ACC and SARC (*P < 0.05; **P < 0.01; ***P < 0.001; ****p < 0.0001; ns, no significant difference).

Tumorigenesis is an outcome of the complex interactions between tumor cells and their microenvironment. We further explored the possible molecular mechanisms of action of *NBPF1* in tumorigenesis and progression by analyzing the association between *NBPF1* and tumor immunity in various cancers. The immunophenotype spans traditional anatomical classifications and reflects the integrated regulatory network of immune cells (16), which is closely related to immune features as well as clinical outcomes. We observed different trends of up and downregulation of *NBPF1* expression in different immune subtypes of a given cancer type, suggesting the potential immunological role of *NBPF1* in certain tumors (**Supplementary Figure 3**). We further analyzed the correlation between *NBPF1* transcription and tumor-infiltrating lymphocytes (TIL) abundance using the TISIDB database (**Supplementary Figure 4A**). Interestingly, *NBPF1* was negatively associated with most immune cells in 30 cancer types, except for KIHC and PAAD, with the negative correlation being particularly evident in ACC. We also observed that the transcription of *NBPF1* in patients with ACC seemed to be adversely linked with stromal, immunologic, and ESTIMATE scores (**Supplementary Figure 4B**). To prevent computational mistakes caused by using a single method and different sets of TIL-tagged genes, we used eight algorithms to calculate the potential association between *NBPF1* transcription and infiltrating stromal and immune cells. Although there was some variation in the results obtained by the eight different algorithms, we noted that at least seven algorithms—CIBERSORT (**Figure 6A**), MCP-counter (**Supplementary Figure 5A**), CIBERSORT-ABS (**Supplementary Figure 5B**), quantTIseq (**Supplementary Figure 5C**), TIP (**Supplementary Figure 5D**), xCell (**Supplementary Figure 6A**), EPIC (**Supplementary Figure 6B**), and TIMER (**Supplementary Figure 6C**)—produced calculations indicating that the proportions of CD8 T cell and macrophage recruitment in ACC patients were adversely linked to *NBPF1* expression. We also observed a positive correlation between *NBPF1* transcription and mast cell infiltration in BRCA (**Supplementary Figures 7A–I**), LUAD (**Supplementary Figures 7J–L**), and KIRC (**Supplementary Figures 7M–O**).

Moreover, we demonstrated that *NBPF1* expression in ACC and SARC was adversely associated with the recruitment of immune cells (**Figure 6B**) involved in the anti-cancer immune response, and the seven steps of the anti-cancer immune response were differentially suppressed in patients with high expression of *NBPF1* (**Figures 6C, D**). Considering the impact of different pathological stages on the tumor microenvironment, we compared the level of immune cell infiltration between the high- and low-*NBPF1* expression groups in patients with ACC with early- (N0) and late-stage (N1) tumors. **Supplementary Figure 8** shows that high expression of *NBPF1* is associated with suppression of immune cell infiltration in patients with early-stage tumors as taken from the ssGSEA algorithm, which is consistent with our previous findings. However, it is not feasible for us to perform the aforementioned analysis on ACC patients with advanced tumors due to the small number of patients with stage N1 ACC (N = 9) and the fact that only one of them was assigned to the *NBPF1*-low expression group.

Additionally, *NBPF1* was found to be negatively correlated with numerous immunomodulators (MHC, chemokines, receptors, and immunostimulators) (**Supplementary Figure 9**) in ACC, SARC, and THCA, which are crucial for the activities of the cancer immunity cycle.

Given the association between *NBPF1* and tumor immunity, we explored the role of *NBPF1* in tumor immunotherapy. We found that the expression of *NBPF1* was mutually exclusive of several immune checkpoints in ACC and SARC (**Supplementary Figure 10**). Likewise, TMB has been identified as a marker of immune checkpoint inhibition (ICI) in the therapeutic response (17); *NBPF1* was negatively correlated with TMB in ACC, THYM, and THCA (**Supplementary Figure 11**). Thus, high levels of *NBPF1* expression may predict unsatisfactory immunotherapeutic outcomes when targeting immune checkpoint genes in these cancers.

### Analysis of the biological functions of *NBPF1* in ACC

**Figure 7:**
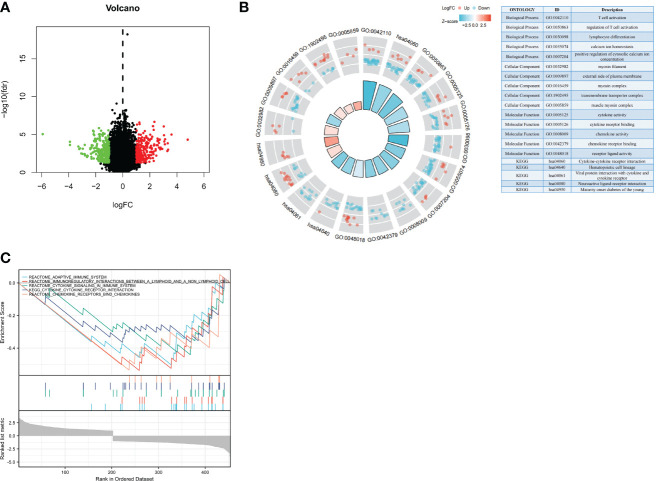
Biological functions of *NBPF1* in ACC. **(A)** Volcano plot showing differentially expressed genes of high- and low-*NBPF1* expression groups. **(B)** GO/KEGG enrichment analysis. **(C)** GSEA enrichment analysis.

Our observations thus far have revealed substantial links between *NBPF1* expression and the prognosis and immunological response of ACC patients. We subsequently identified DEGs between *NBPF1* groups with elevated or lowered expression levels. According to our generated volcano plot, there were 510 DEGs identified between the elevated/low expression ACC groups, including 235 elevated genes and 275 dysregulated genes (**Figure 7A**). We then conducted GO/KEGG enrichment analysis based on DEGs; DEGs were abundant in cellular components, including the myosin filament, external side of the plasma membrane, myosin complex, transmembrane transporter complex, and muscle myosin complex. We also observed that the DEGs were involved in certain molecular functions such as cytokine activity, cytokine receptor binding, chemokine activity, chemokine receptor binding, and receptor–ligand activity. The GO analysis of the biological processes revealed that DEGs were recruited for T-cell activation, regulation of T-cell activation, lymphocyte differentiation, calcium ion homeostasis, and positive regulation of cytosolic calcium ion concentration (**Figure 7B**).

Through GSEA analysis, we also found that DEGs were mainly involved in the regulation of immune responses in ACC patients, including that of the adaptive immune system, immunoregulatory interactions, cytokine signaling, cytokine–receptor interactions, and chemokine receptor binding (**Figure 7C**). This is also in line with our previous findings that *NBPF1* plays a vital role in ACC by reducing the immunological response of the TME. Meanwhile, GSEA analysis was carried out to confirm the putative signaling pathways of *NBPF1* in the external (GEO) dataset, and the results suggested that genes relevant to *NBPF1* were negatively associated with cytokine–cytokine receptor interactions, primary immunodeficiency, and the toll-like receptor signaling pathway (**Supplementary Figure 12**). The particular enrichment results have been included in **Supplementary Table 4**.

### Construction and validation of an IRS

Using Venn analysis, we identified 65 common IDEGs (**Supplementary Figure 13**), and a univariate Cox regression assessment was used to yield 27 differentially expressed IRPGS (**Table 3**), which were found to be highly linked to overall survival among ACC samples (P<0.05). A LASSO regression assessment was then performed on these 27 IRPGS, and 13 significant IRPGS were employed to build an IRS based on the training cohort.

**Table 3:** Immune-related prognostic genes.

ID	HR	HR.95L	HR.95H	p-value
*LHB*	1.458850505	1.232480891	1.726797399	1.14E−05
*TRH*	1.403178776	1.154020459	1.706131517	0.000683269
*GDF10*	1.221115562	1.083656455	1.376011012	0.001043585
*TACR1*	0.669148234	0.525980068	0.851285793	0.001072516
*SLPI*	0.848338361	0.767637826	0.937522814	0.001260143
*SEMG1*	0.801994407	0.698386968	0.920972267	0.001769449
*GALR3*	1.986727577	1.271963499	3.103144444	0.002550417
*BMP7*	1.158400958	1.051979387	1.275588472	0.002784401
*CXCL3*	1.286557836	1.08640339	1.523587907	0.003494512
*PRL*	1.413770442	1.116346111	1.790436533	0.004062832
*GREM2*	1.158412057	1.047163719	1.281479171	0.00430928
*NBPF1*	1.517811685	1.136112822	2.027749592	0.004750912
*EPO*	1.342667577	1.093876885	1.648043071	0.004830437
*CD1C*	0.701180131	0.536035053	0.917204152	0.009578458
*MC3R*	0.548034603	0.340505907	0.882046154	0.013254159
*CD1E*	0.65000458	0.461047961	0.916403477	0.013967025
*CD40LG*	0.651052207	0.45856289	0.924342082	0.016399279
*CR2*	0.607157633	0.403101884	0.914509225	0.016958992
*PTGDR*	0.736631964	0.569124741	0.953440629	0.020223299
*CXCR6*	0.798829526	0.658559347	0.968976622	0.022616545
*QRFP*	1.551747101	1.060287574	2.271005646	0.02374468
*FGF21*	1.207172815	1.024211611	1.422817502	0.024752283
*REG3G*	0.83150364	0.705533934	0.979964634	0.027705994
*GDF2*	1.35809289	1.028829298	1.792733063	0.030727185
*CD244*	0.748818991	0.572438871	0.979545431	0.034791855
*NCR3*	0.685470513	0.481192308	0.976469941	0.036451186
*GRAP2*	0.739310755	0.556603599	0.981992198	0.037030003


$$\begin{array}{l} Risk\;score=(0.2071\;\times \;expression\;value\;of\;LHB)+(0.0831\;\times \;expression\;value\;of\;GDF10\;)+\\ (-0.0819\;\times \;expression\;value\;of\;TACR1)+(-0.0241\;\times \;expression\;value\;of\;SEMG1)+(0.0067\;\times \;\exp ression\;value\;of\;GALR3)+(0.0361\;\times \;expression\;value\;of\;BMP7)+(0.3516\;\times \;\exp ression\;value\;of\\ CXCL3)+(0.1651\;\times \;\exp ression\;value\;of\;PRL)+(0.1379\;\times \;\exp ression\;value\;of\;NBPF1)+(-0.0313\times \;\exp ression\;value\;of\;MC3R)+(-0.1194\;\times \;\exp ression\;value\;of\;CR2)+(0.0096\;\times \;expression\;value\;of\\ FGF21)+(-0.0227\;\times \;expression\;value\;of\;GRAP2) \end{array}$$

**Figure 8:**
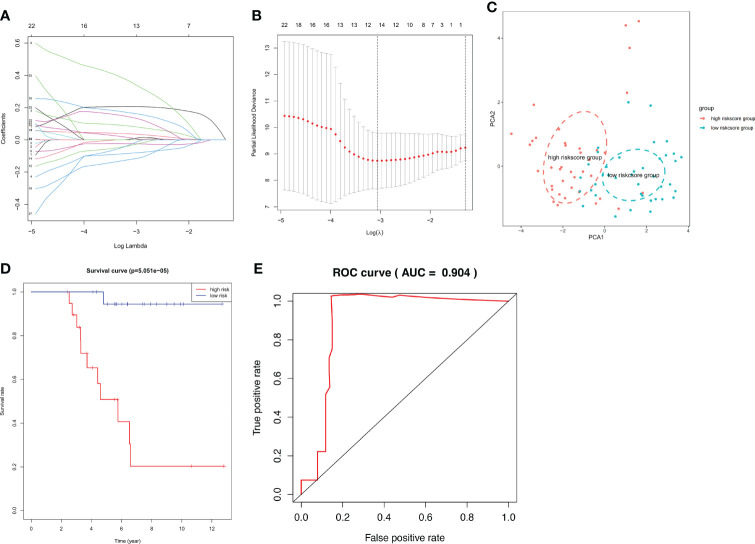
IRS RNA-expression profiles. **(A, B)** LASSO coefficient spectrum of the 13 selected genes from the TCGA training cohort. **(C)** Principal Component Analysis (PCA) stratification of TCGA-ACC patients into low- and high-risk groups. **(D)** Kaplan–Meier curve showing that the survival rate of the low-risk group was significantly greater than the high-risk group in the TCGA training cohort. **(E)** ROC curve showing the predictive efficiency of the risk scores.

The risk score of each sample in the training cohort was calculated according to the prognostic model, and the median risk score in the training cohort was set as the cut-off value (8.5) to divide the ACC training patients into high- and low-risk groups. Km analysis revealed that the high-risk group had a shorter OS (5.051e−05) than the low-risk group. ROC curves demonstrated that the immune risk signature provided an excellent level of prediction accuracy for ACC prognosis in the training group (**Figures 8A–E**). Next, the same coefficients and cut-off values were applied to the test group. The same pattern was shown by the generated Km curves, and the ROC curve also confirmed the high prediction accuracy of the risk score for ACC prognosis in the testing group (**Supplementary Figure 14**). However, disappointingly, accurate prediction could not be achieved (**Supplementary Figure 15**) when we used the same cut-off value in the validation cohort

**Figure 9:**
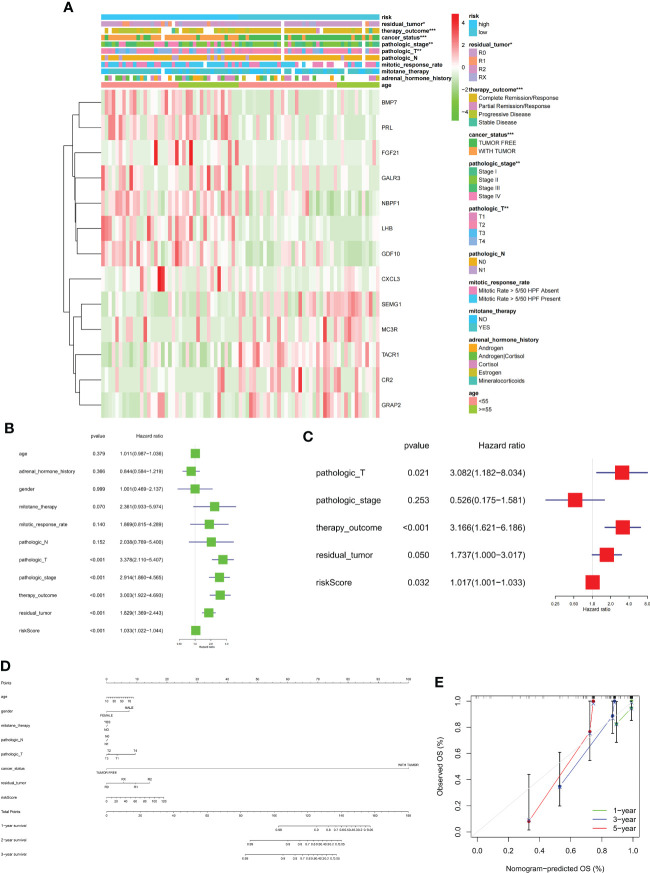
**(A)** Heatmap comparison of several clinical factors between low- and high-risk groups. **(B, C)** Univariate Cox regression (green) and multivariate Cox regression (red) showing that the risk signature is an independent prognostic factor for ACC patients. **(D)** Normogram and **(E)** calibration curve prediction of the probability of 1-,2-,3-years OS for ACC patients (*p < 0.05, **p < 0.01, ***p < 0.001).

The clinical prognostic monitoring of patients with ACC depends largely on various factors, such as clinicopathological characteristics. Our univariate combined multivariate regression analysis showed that the risk score is an independent indicator, so we inserted the immune risk signature into our ACC prognostic analysis and constructed a nomogram to predict the overall survival rate of an individual at 1, 2, and 3 years. The calibration plots showed that the nomogram performed well in predicting the 1-, 2-, and 3-year OS compared with an ideal model. Moreover, the heatmap showed that high-risk signatures were significantly associated with undesirable clinicopathological status, including pathological stage and high T classification (**Figures 9A–E**).

### Association between IRS and immunological characteristics

**Figure 10:**
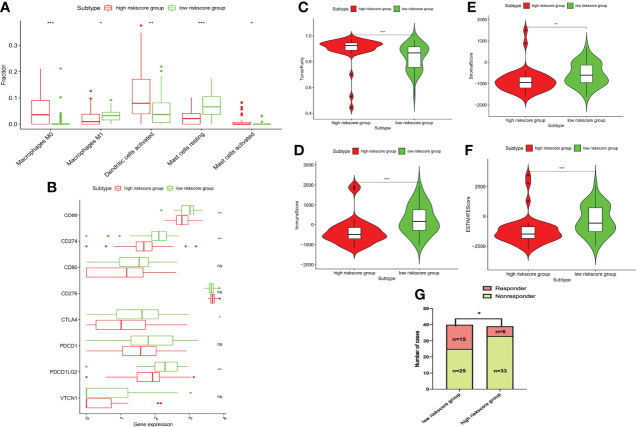
Immune characteristics among different risk groups. **(A)** Bar chart showing the immune infiltrating cells that are significantly different between the two risk groups. **(B)** Tumor purity. **(C)** Immune, **(D)** stromal, and **(E)** ESTIMATE scores. **(F)** Bar chart showing the immune checkpoints that are significantly differentially expressed between the two risk groups. **(G)** Correlation between risk score and the clinical response to immunotherapy (*P < 0.05, **P < 0.01; ***P < 0.001) ns, no significant difference.

In addition to its prognostic value, the *NBPF1*-derived immune risk signature is highly associated with TME in TCGA-ACC patients. As shown in **Figure 10A**, high immune risk can facilitate infiltration of M1 macrophages and resting mast cells while reducing levels of M0 macrophages and activated dendritic cells compared to the low-risk group. Additionally, stromal, immune, and ESTIMATE scores were significantly lower in the high IRS group, suggesting that the process of immune infiltration and the formation of multiple components were significantly inhibited in the high-risk population (**Figures 10B–E**). Remarkably, we noticed that the clinical response to immunotherapy was stronger in the low IRS population of the TCGA-ACC cohort. It was found that the expression levels of several immune checkpoints, such as *PDCD1LG2*, *CTLA4*, *CD274*, and *CD86*, were significantly lower in the high-risk population than in the low-risk population (**Figures 10F, G**).

### Drug sensitivity

**Figure 11:**
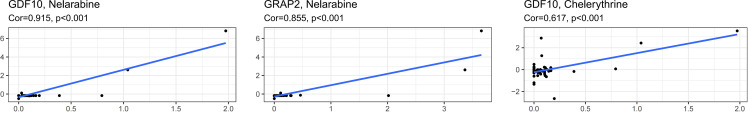
Correlation between IRS RNA-expression and drug sensitivity as derived from the NCI-60 cancer cell set.

Finally, we assessed the relationship between the 13 IRPGS expression levels and the drug activity of anti-tumor drugs in the NCI-60 cancer cell set and found that, as shown in **Figure 11**, the sensitivity of nelarabine was increased by GDF10 (Cor = 0.915, p<0.001) and GRAP2 (Cor = 0.855, p<0.001), and the upregulation of GDF10 also enhanced the drug sensitivity of chelerythrine (Cor = 0.617, p<0.001).

## Discussion

Our study employed publicly financed cancer genomics programs and archives to decipher the landscape of various tumor types based on *NBPF1* expression to determine its likely function in tumorigenesis. In addition to multilevel data comparison of *NBPF1* across multiple cancers, we examined the links between *NBPF1* expression and clinical manifestations, prognosis, TIME, TMB, immunotherapeutic effectiveness, genetic alteration, function labeling, and enrichment. This comprehensive pan-cancer study revealed the probable biological function of *NBPF1* in the development, progression, and clinical prognosis of various human cancers.

In our study, the expression of *NBPF1* was significantly downregulated in 10 cancer types in TCGA, while others showed higher expression when compared with paired adjacent normal tissues. This heterogeneity may reflect different underlying functions and mechanisms of *NBPF1* across multiple cancer types. Ma et al. reported that Chinese colorectal cancer patients with lower *NBPF1* levels showed worse survival rates than those with higher expression levels (18), which is consistent with our finding that *NBPF1* is a prognostically relevant protective factor in most tumor types. Interestingly, we found that in a small subset of cancer types, high expression of *NBPF1* leads to worse survival outcomes. Notably, both KM analysis and univariate Cox regression assessment suggested that higher expression of *NBPF1* was associated with worse survival in patients with ACC. ACC is a rare human malignancy with heterogeneous clinical features and a poor prognosis (19). It has a complex pathogenesis involving multiple aberrant signaling pathways and lacks robust biomarkers to predict the prognosis of patients with ACC. To our knowledge, our study is the first to indicate that *NBPF1* expression may be utilized as a prognostic factor in ACC, referring to univariable and multivariable Cox regression. Furthermore, existing research has revealed that NBPF family genes show a high degree of copy number fluctuation in various human diseases, which may be implicated in developmental disorders, craniofacial dysmorphism, and early tumorigenesis of neuroblastoma (20, 21). Subsequently, we further investigated *NBPF1* alterations using the cBioPortal database and observed that *NBPF1* showed the greatest prevalence of copy number alterations in patients with cholangiocarcinoma. Given the probable role of NBPF family genes in pathological processes, it is worth examining the role of *NBPF1* gene variation, particularly copy number alterations, as a cancer sensor in patients with cholangiocarcinoma. We found that amplification was the most prevalent kind of alteration in ACC patients, and, using the GSCA database, we found that *NBPF1* expression was shown to be inversely linked with methylation levels in patients with ACC. Moreover, those with hypomethylation of *NBPF1* had a worse prognosis. These findings indicate that *NBPF1* is a promising prognostic biomarker for ACC and that DNA copy number amplification and methylation may be the two underlying drivers of *NBPF1* dysregulation in ACC.

Multiple studies have revealed that the tumor microenvironment not only has a significant impact on tumor development but also plays a key role in immune evasion and treatment resistance. Tumor immune infiltrating cells, an important component of the tumor microenvironment, determine the immune landscape. We employed different methods to evaluate the association between *NBPF1* and TIL abundance and observed that *NBPF1* gene expression is strongly linked to the degree of mast cell infiltration in patients with LUAD and BRCA. According to a review by Aponte-López et al. (22), high mast cell infiltration has been linked to a better prognosis in non-small cell lung cancer and BRCA in several clinical trials, which is consistent with our prognostic analysis. Therefore, we propose that *NBPF1* may work in BRCA and LUAD patients by modulating the number of anti-tumor mast cells in the tumor microenvironment, which further contributes to a better prognosis. In contrast, CD8 T cells were significantly reduced in ACC patients with high *NBPF1* expression. Consistent with previous studies, the abundance of CD8+ T cells had an anti-tumor effect and was linked with a positive prognosis, tumor size, and staging in most types of cancers. The anti-cancer immune response can be conceptualized into seven steps: release of cancer cell antigens (Step 1), cancer antigen presentation (Step 2), priming and activation (Step 3), trafficking of immune cells to tumors (Step 4), infiltration of immune cells into tumors (Step 5), recognition of cancer cells by T cells (Step 6), and killing of cancer cells (Step 7) (23). Given the intricacy of the mechanisms underlying the anti-cancer immune response, we assessed immune activation processes and immune infiltration among 33 cancer types and found that *NBPF1* may inhibit the activation of anti-tumor immunity, particularly in ACC and SARC. GSEA of *NBPF1* also revealed that immune-regulation-relevant pathways were enriched in ACC. In recent years, a growing number of studies have found that decreased TIL levels are associated with higher TNM and AJCC staging and better OS and PFS in patients with ACC (24). Drawing on the findings of the preceding investigation, we hypothesized that *NBPF1*-mediated inhibition of TIL infiltration might be a prime driver of its oncogenic effect in ACC. Despite the fact that the relationship between TME and immunotherapy efficacy has received comparatively little investigation in ACC, T-cell infiltration is required for successful immunotherapeutic implementation. It has also been found that *NBPF1* may regulate the expression of specific immune checkpoint genes. Our study presents evidence for an association between *NBPF1* and TMB. These results imply that future research efforts should concentrate on modifying the TME by targeting *NBPF1*, provide insight into the potential of *NBPF1* expression to predict the effect of immunotherapy, and stratify patients for the selection of those who should follow immune checkpoint blockade treatment.

Furthermore, we constructed and validated a novel immune risk signature and demonstrated that it is involved in the prognosis, progression, and TME of patients with ACC. The prognosis of the high-risk population, which is substantially linked to unsatisfactory clinical staging and a higher T classification, is poorer than that of the low-risk population. Although in the external dataset, this cut-off of the risk score that we have established did not distinguish between two different prognostic ACC populations, we believe that this might be the result of batch effects brought on by various sequencing platforms and methodologies. We believe that specific cut-offs should be established for patients with different platforms or laboratory origins. Nomograms integrated with immune risk signatures and clinical features were developed to monitor the survival rates of patients with ACC. The application of the nomogram in clinical practice may provide more detailed information on survival. Additionally, in this study, the risk signature extensively affected the activities of various immune cells in the TME. M0 macrophages can polarize into M1 and M2 macrophages in different environments, and evidence accumulated from many cancer models demonstrated that M1 macrophages possess inflammation-promoting characteristics, which activate cytotoxic anti-tumor mechanisms (25). According to Huang et al., the degree of M0 macrophage infiltration was significantly higher in metastatic ACC than in non-metastatic patients (26). Notably, the immune risk may lead to the suppression of M1 macrophage infiltration and the promotion of M0 infiltration, which suggests that high immune risk may contribute to tumor immune escape and poor prognosis in patients with ACC. In addition, high immune risk can also suppress the expression of immune checkpoint markers and is associated with a failed immunotherapy response. Inspired by the results from other cancer models, the efficacies of the PD-L1 inhibitors pembrolizumab, nivolumab, and avelumab were evaluated in a number of patients with advanced ACC. However, most of these trials have shown a poor therapeutic response to immune checkpoint blockade (24, 27, 28). The immune risk profile identifies molecular alterations among immunotherapy responders and non-responders, which may contribute to improving patient recruitment in future trials.

To the best of our knowledge, this is the first study to explore the specific role of *NBPF1* in pan-cancer prognostic relevance and cancer immune regulation, particularly in ACC. However, our study has certain limitations. First, more basic experiments are required to determine the functional mechanisms of *NBPF1*. Second, all records in our study were collected from public sources and need to be validated further in prospective clinical studies.

## Conclusion

Our study explored the role of genetic and epigenetic changes in *NBPF1* across generalized carcinoma for the first time. We discovered that *NBPF1* expression differed among tumor tissues and was linked to clinical outcomes across different cancers. Particularly in patients with ACC, *NBPF1* and related immune risk genes may impact tumorigenesis, the tumor microenvironment, and anti-tumor drug selection. Our study provides a basis for further investigation of the role of *NBPF1* in regulating the immune microenvironment and offers novel ideas for developing anti-*NBPF1* immunotherapy for patients with ACC.

## Data availability statement

The original contributions presented in the study are included in the article/**Supplementary Material**. Further inquiries can be directed to the corresponding authors.

## Author contributions

LL and YH designed the study and performed the analysis. SC and YT prepared and revised the manuscript. JW and LQ finalized the manuscript. All authors contributed to the article and approved the submitted version.

## Funding

The study received support from the Capital’s Funds for Health Improvement and Research (CFH-2020-1-4014) and the Beijing Key Clinical Specialty for Laboratory Medicine-Excellent Project (No. ZK201000).

## Conflict of interest

The authors declare that the research was conducted in the absence of any commercial or financial relationships that could be construed as a potential conflict of interest.

## Publisher’s note

All claims expressed in this article are solely those of the authors and do not necessarily represent those of their affiliated organizations, or those of the publisher, the editors and the reviewers. Any product that may be evaluated in this article, or claim that may be made by its manufacturer, is not guaranteed or endorsed by the publisher.
