# Supplementary material for: Oncogene or tumor suppressor gene: An integrated pan-cancer analysis of NBPF1
Source: Front Endocrinol (Lausanne). 2022 Aug 17;13:950326. doi: 10.3389/fendo.2022.950326 (PMC9428449; doi:10.3389/fendo.2022.950326)
Supplement: Supplementary file 1 [file Table_1.docx]

Supplementary Material

# Supplementary Figures


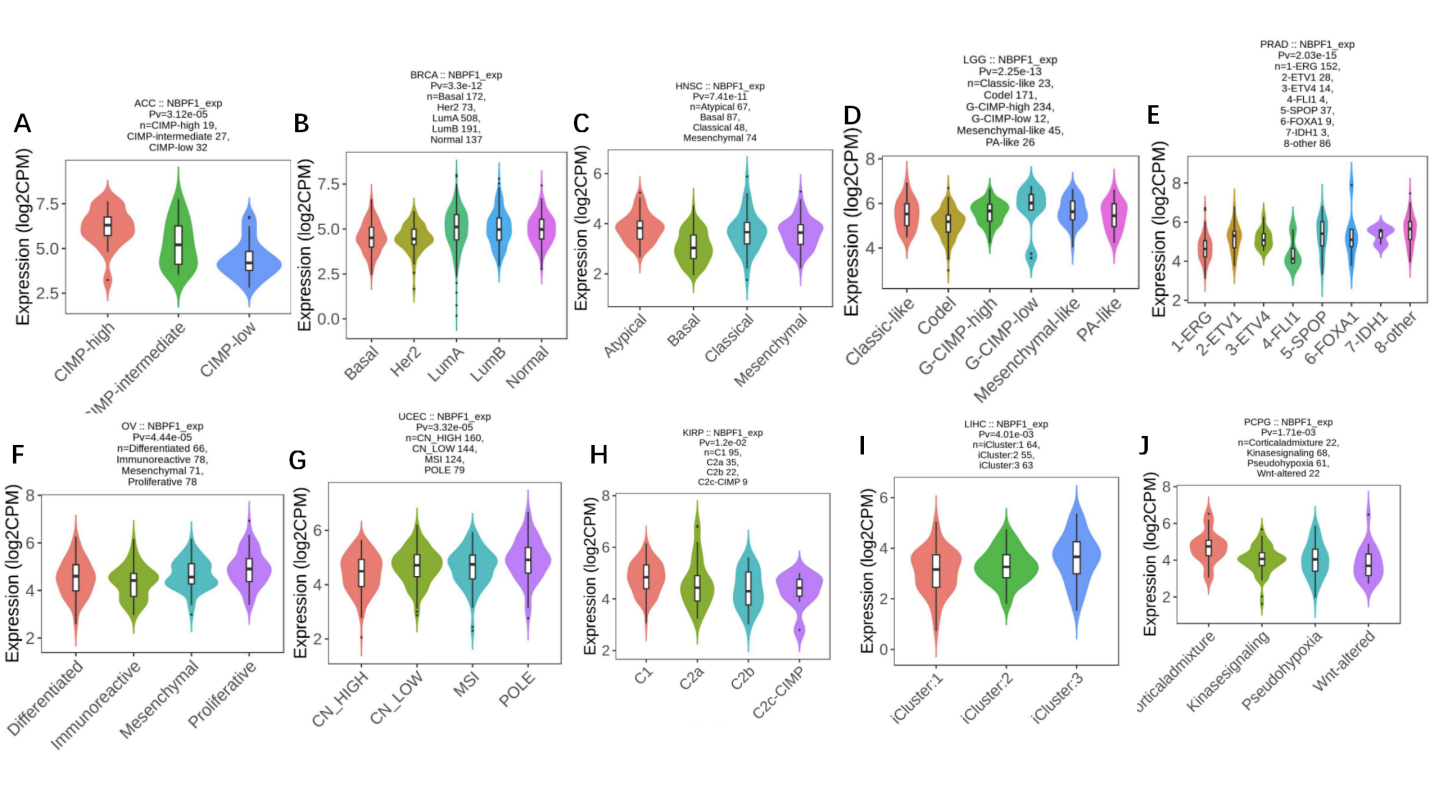


**Supplementary Figure 1.** Expression levels of *NBPF1* in specific cancer subtypes.


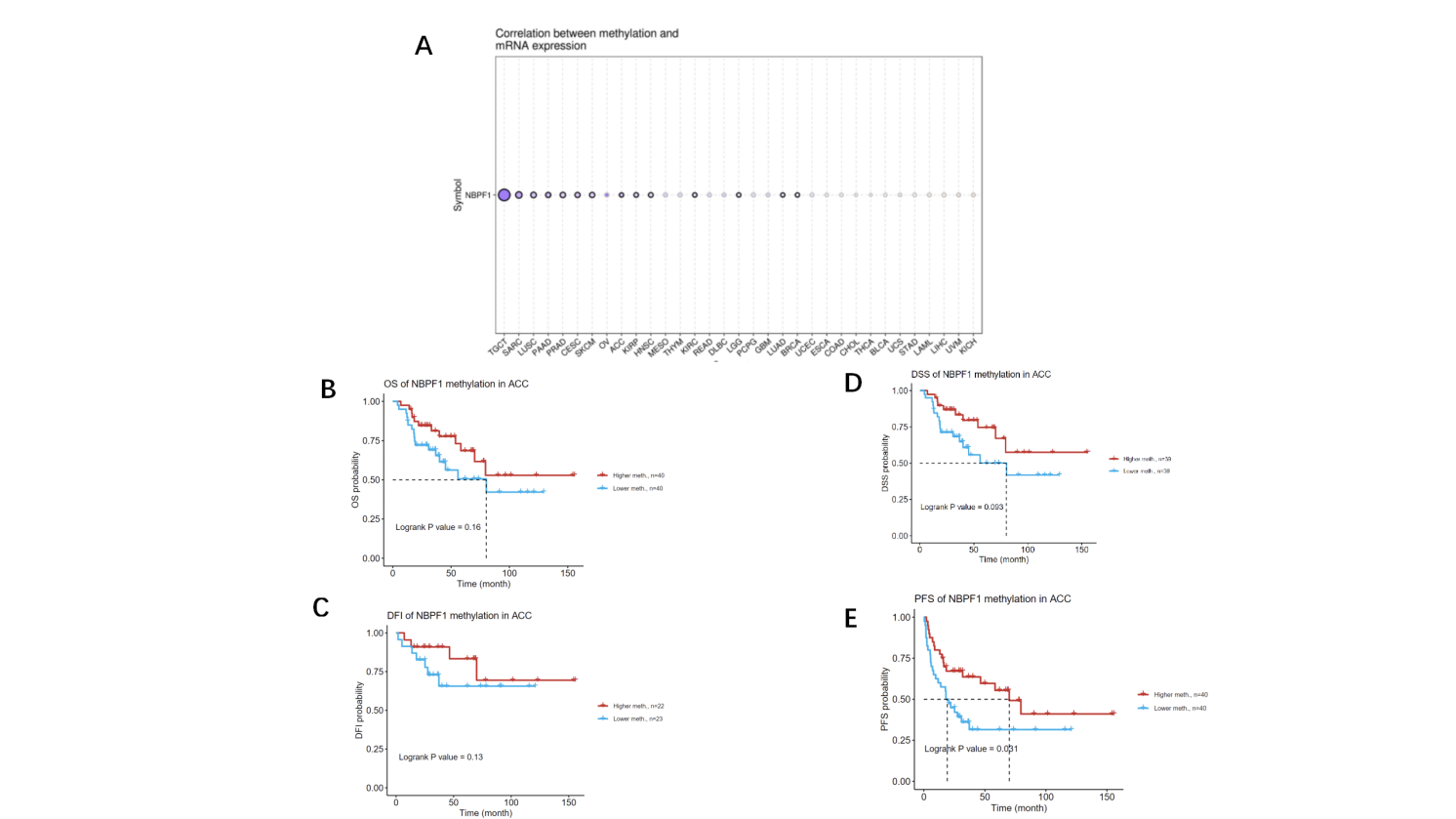


**Supplementary Figure 2.** (A) Correlation between *NBPF1* expression levels and DNA methylation. (B) Correlation between *NBPF1* methylation and OS, PFS, DSS, and DFI in ACC patients.


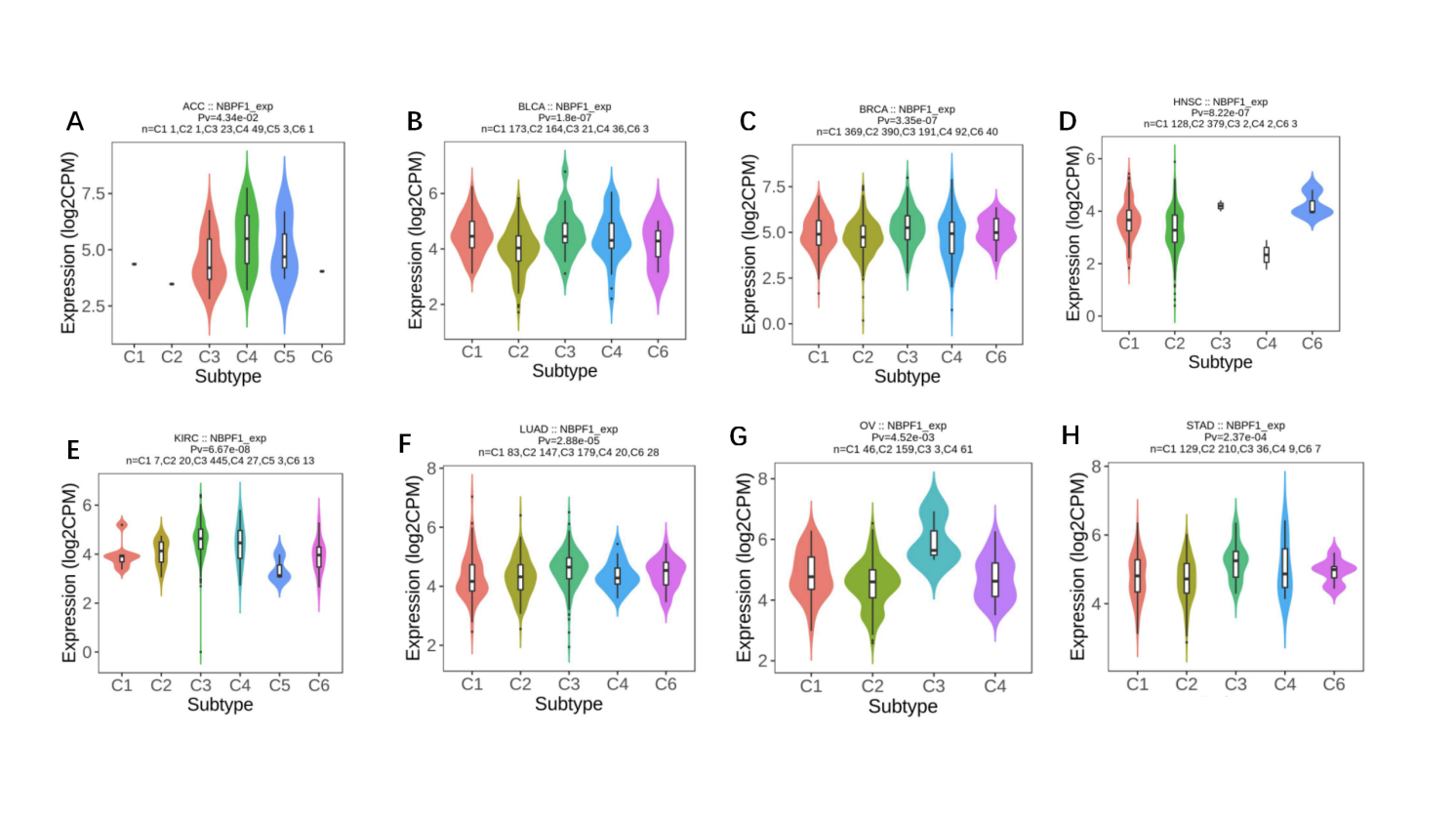


**Supplementary Figure 3.** Expression levels of *NBPF1* in specific cancer immune subtypes.

**
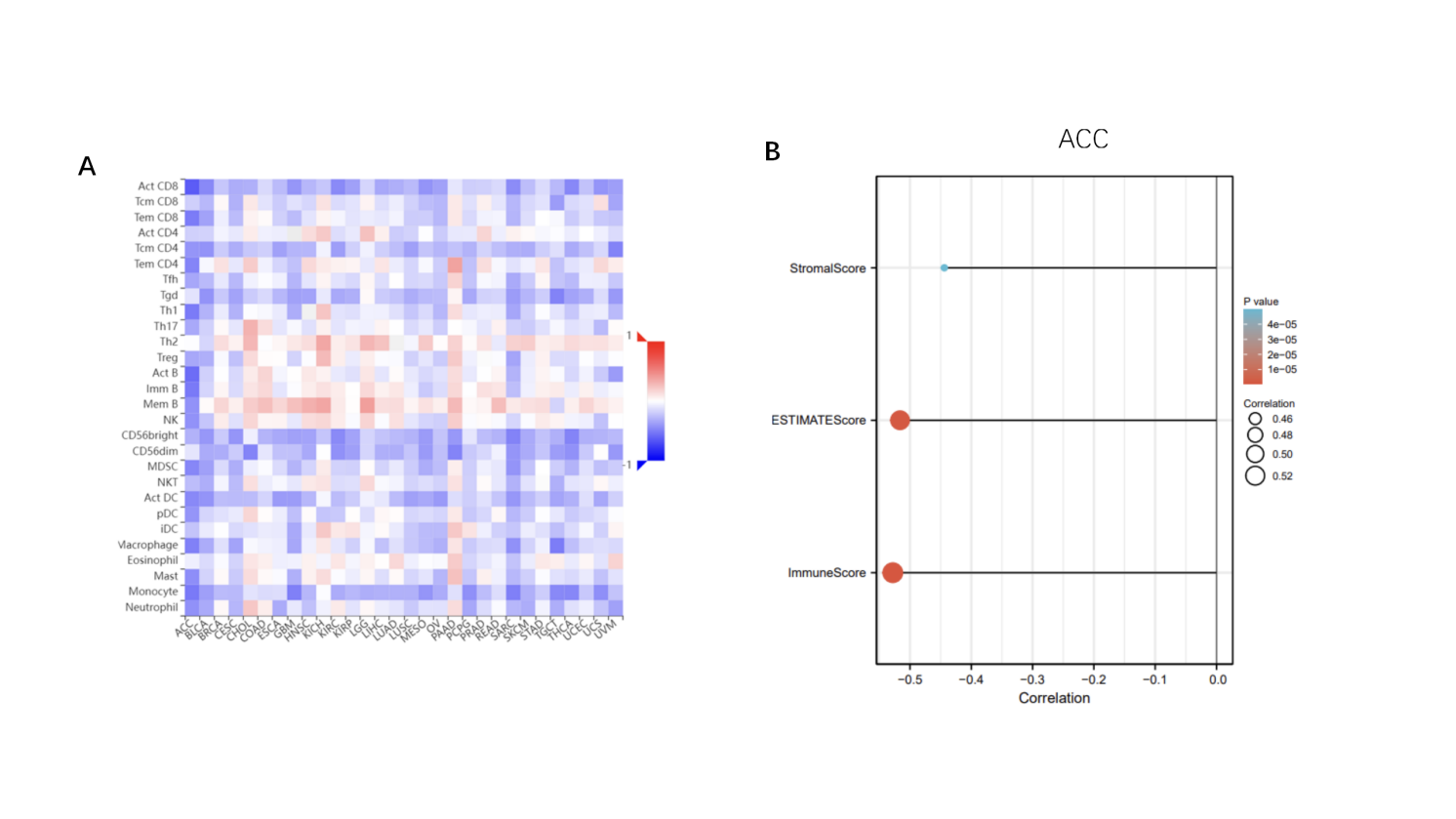
**

**Supplementary Figure 4.** Correlation between *NBPF1* expression and (A) immune cell infiltration via TISDB, (B) stromal score, ESTIMATE score, and immune score.


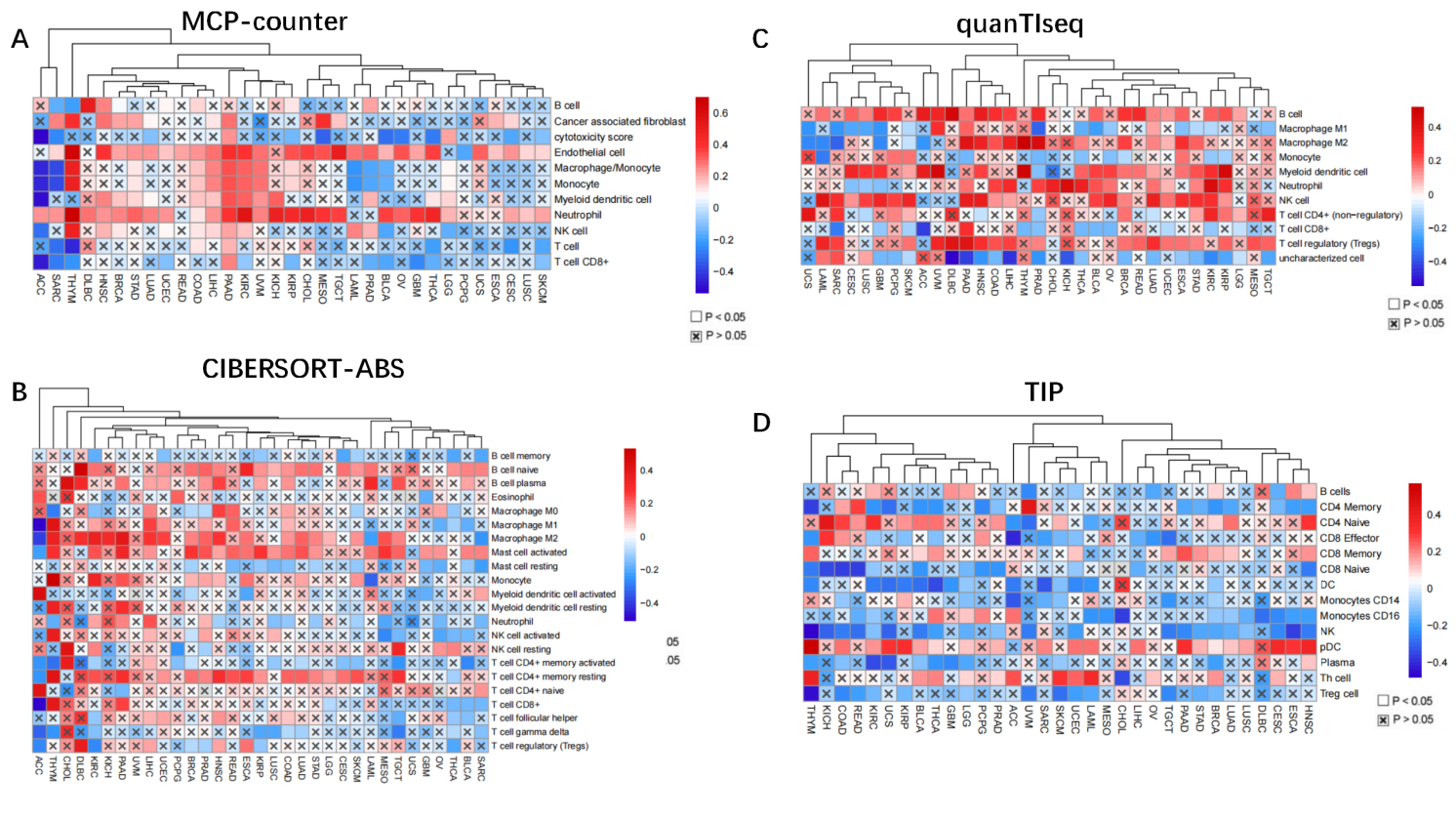


**Supplementary Figure 5.** Correlation analysis between *NBPF1* expression and the abundance of tumor-infiltrating lymphocytes in human cancers via (A) MCP-counter, (B) CIBERSORT-ABS, (C) quanTIseq, (D) TIP algorithms.

**
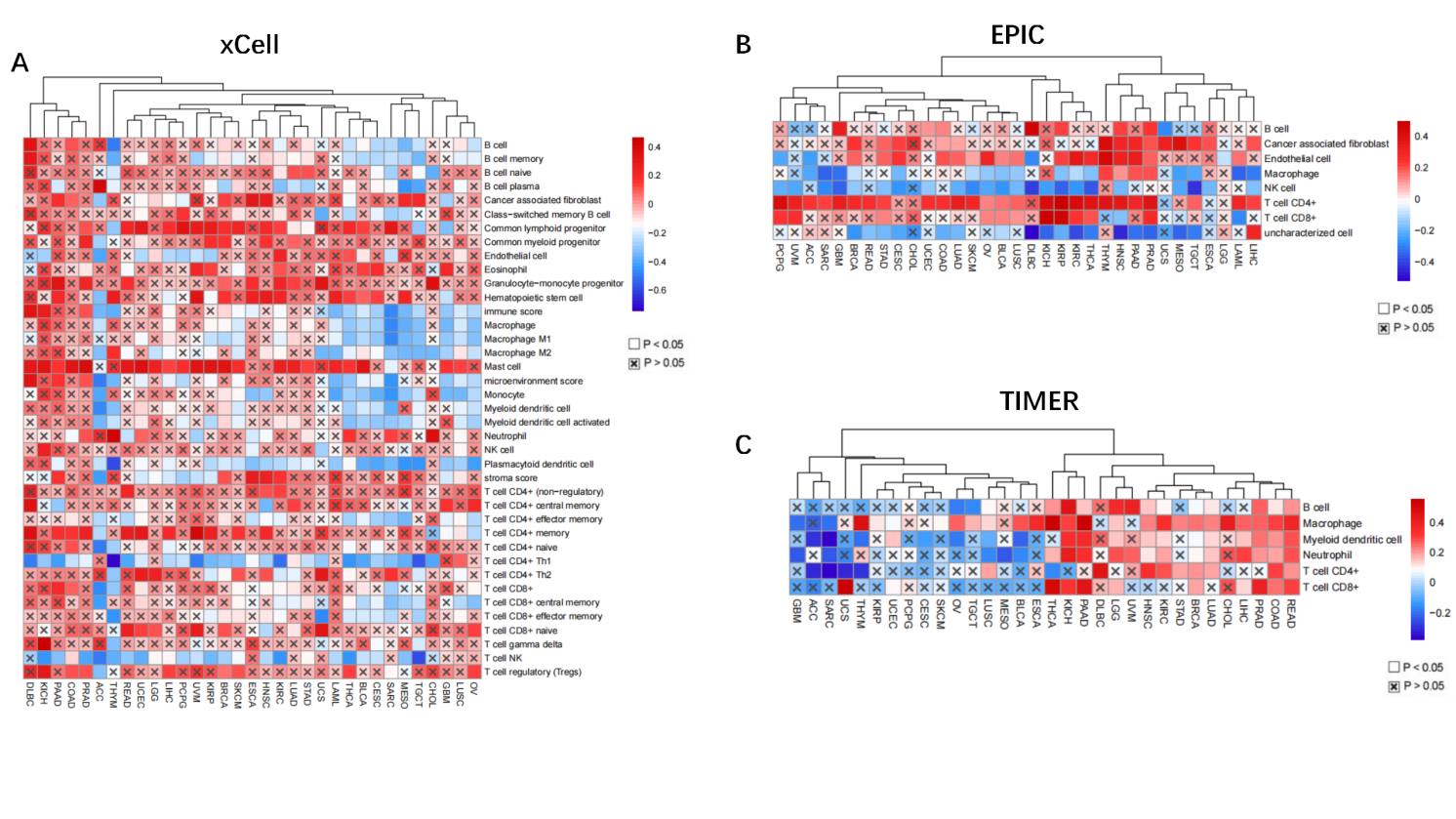
**

**Supplementary Figure 6.** Correlation analysis between *NBPF1* expression and the abundance of tumor-infiltrating lymphocytes in human cancers via (A) xCell, (B) EPIC, (C) TIMER algorithms.

**
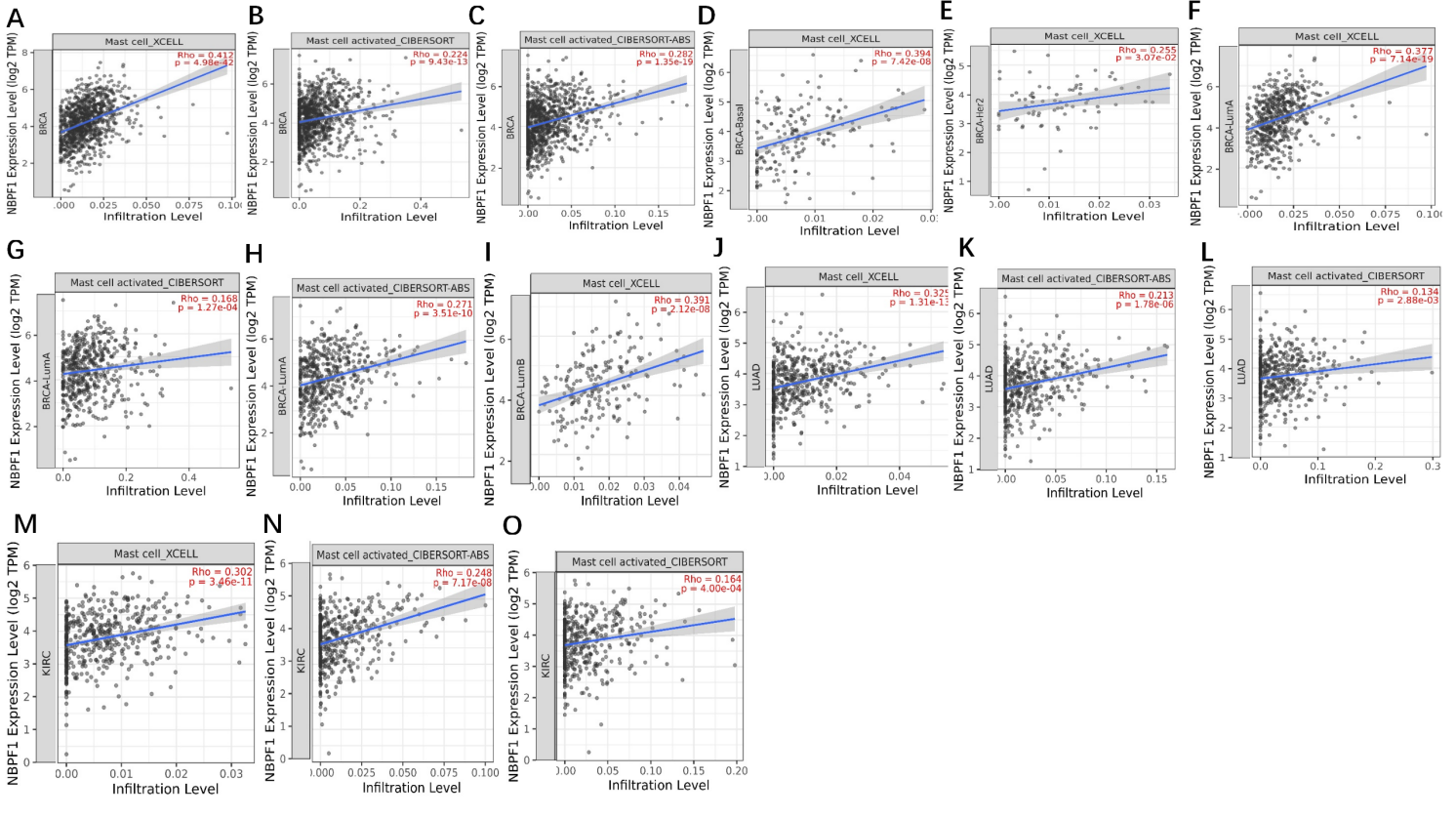
**

**Supplementary Figure 7.**


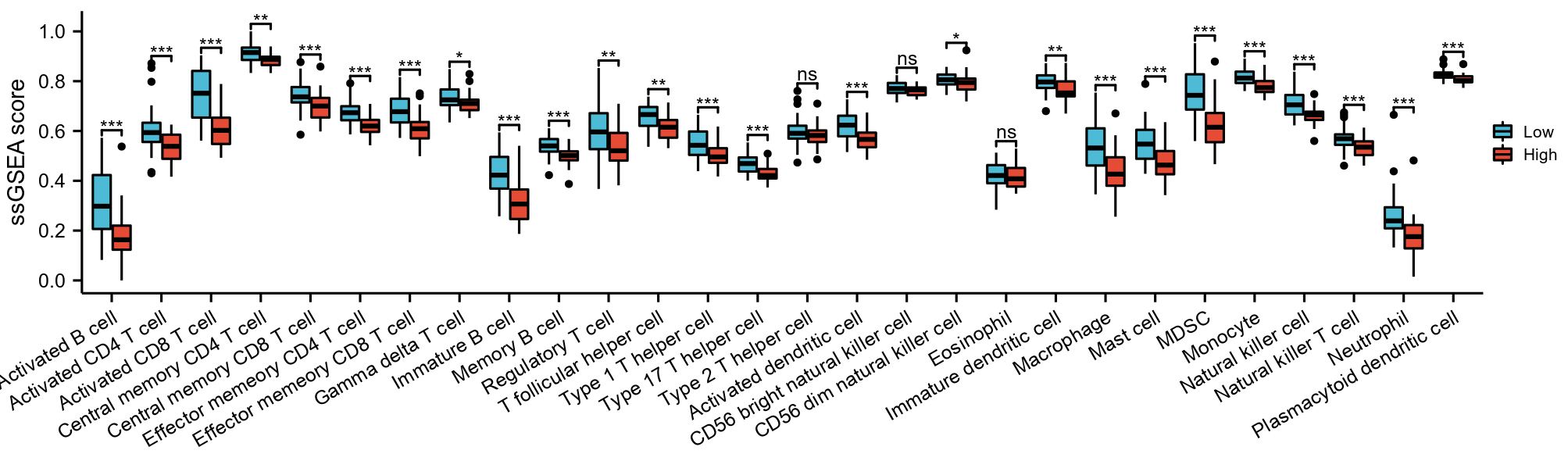


**Supplementary Figure 8.** Differences in tumor-associated immune cell infiltration between high *NBPF1* expression and low *NBPF1* expression groups in ACC patients with early-stage tumors.


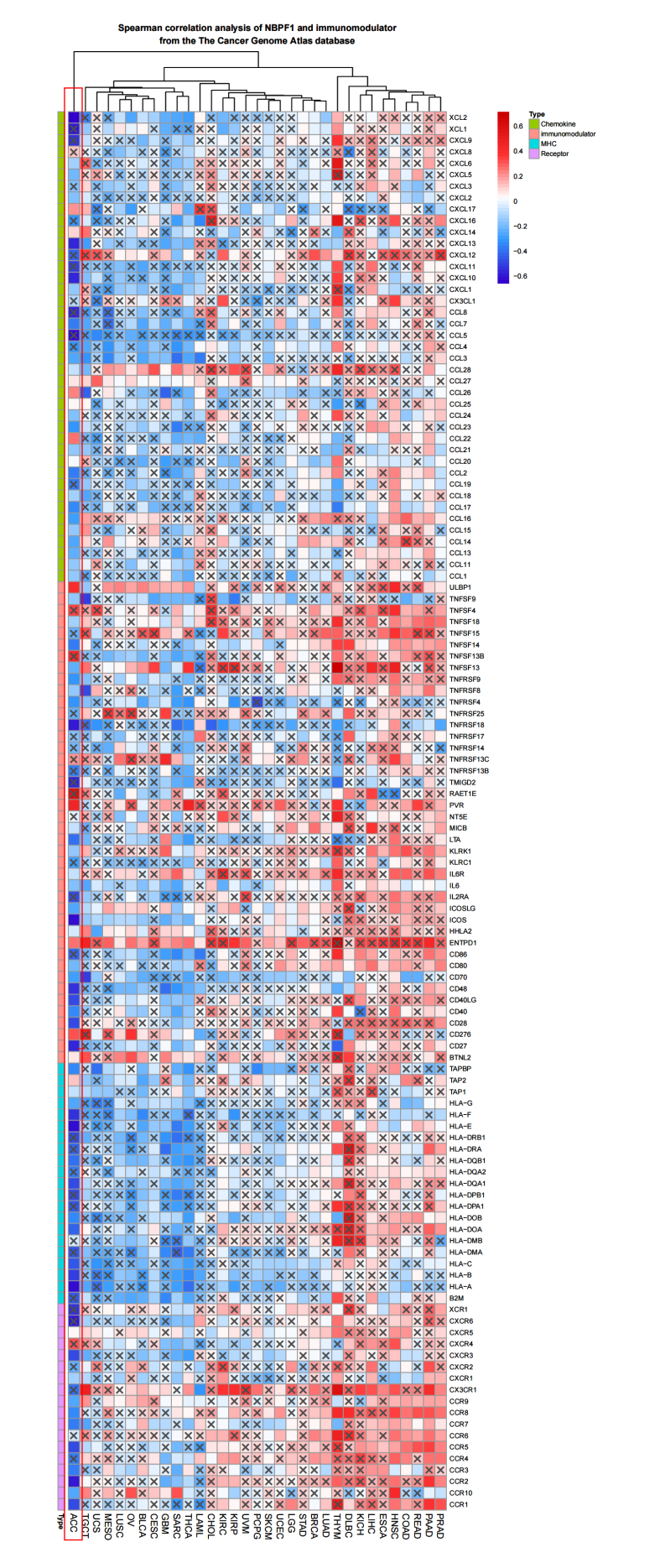


**Supplementary Figure 9.** Correlation between *NBPF1* expression and immunomodulators.

**
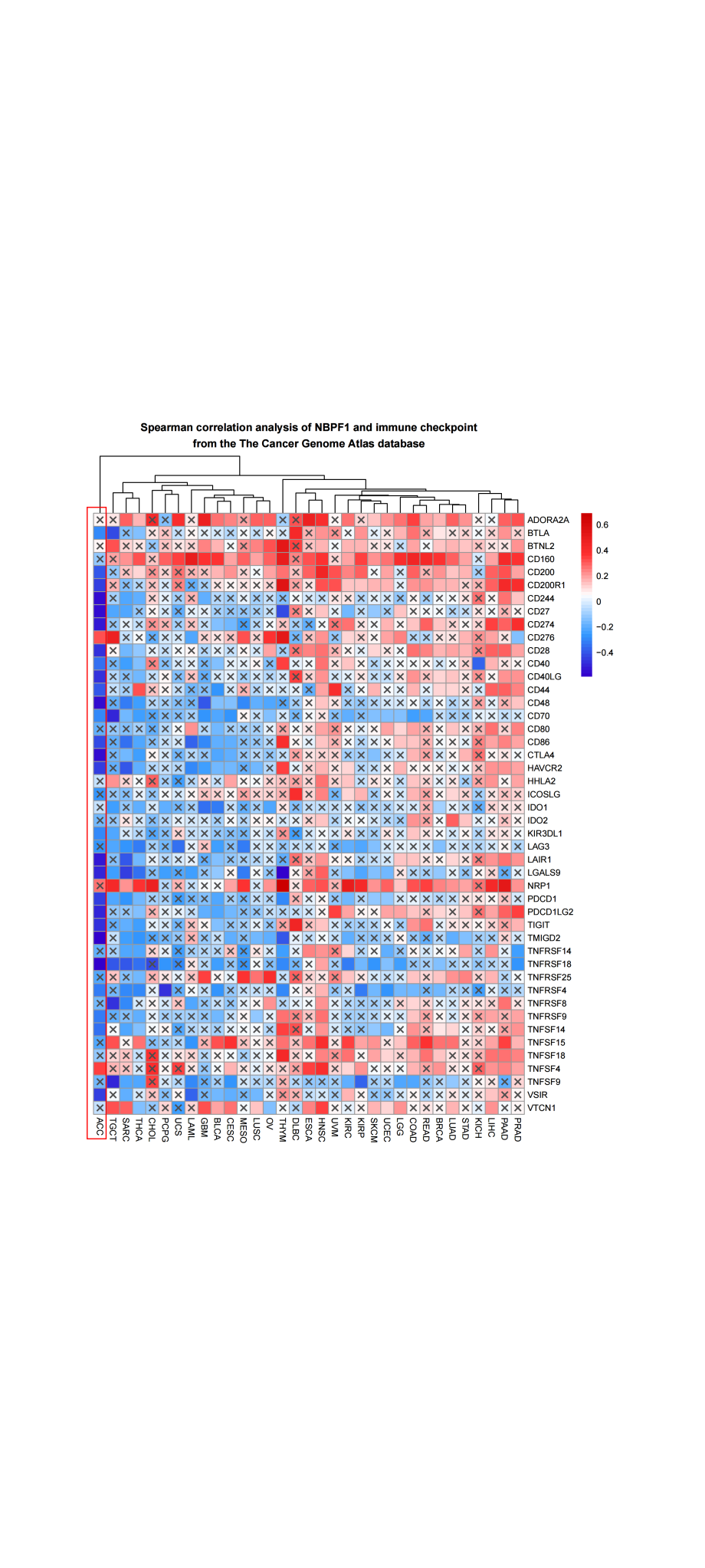
**

**Supplementary Figure 10.** Correlation between *NBPF1* expression and immune checkpoints.


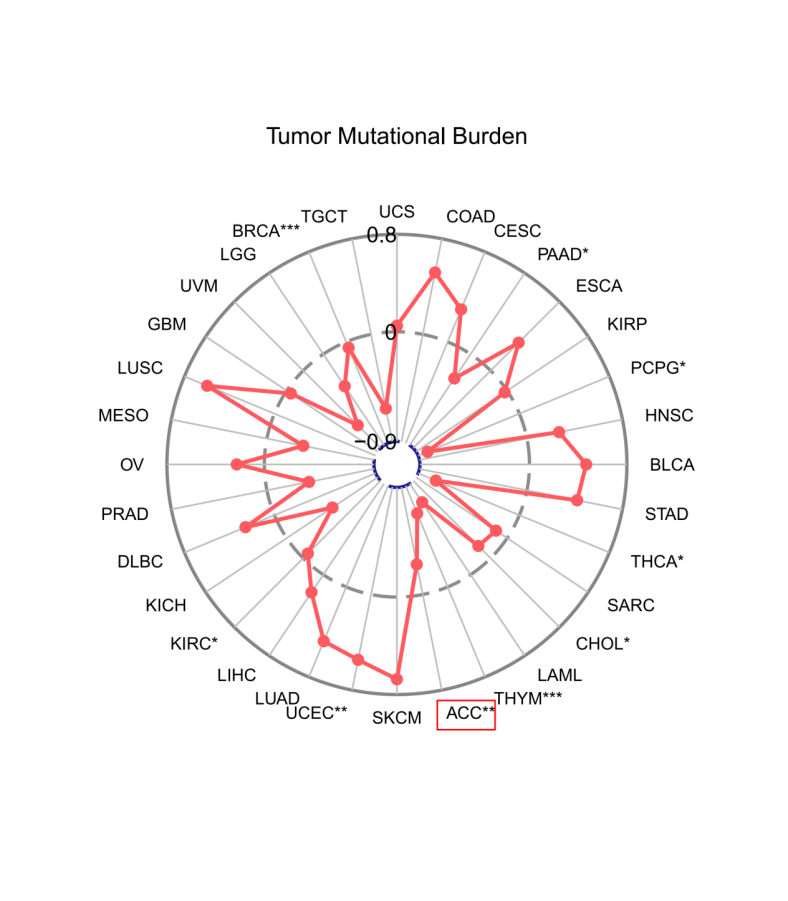


**Supplementary Figure 11.**


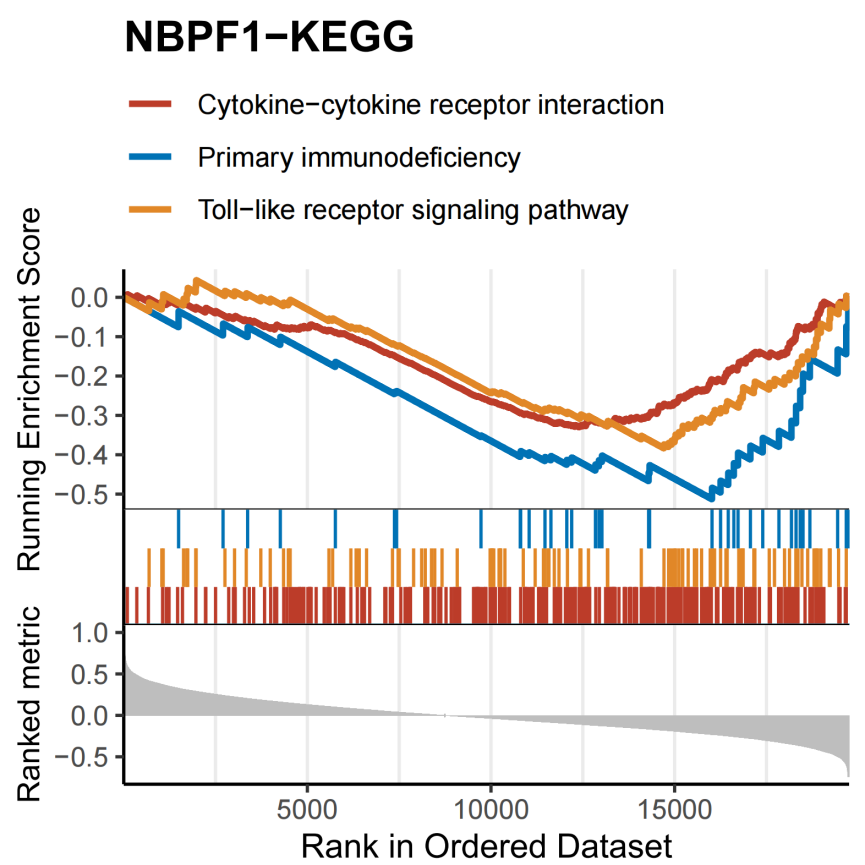


**Supplementary Figure 12.** GSEA enrichment *NBPF1*-related signaling pathway in the GEO dataset.


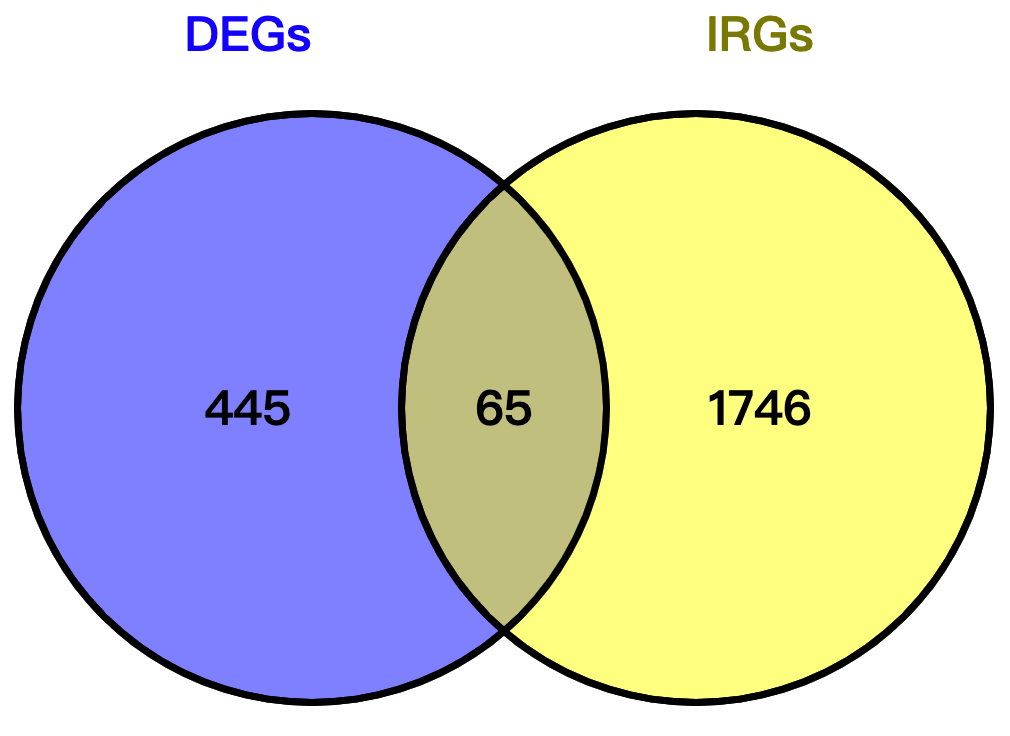


**Supplementary Figure 13.** Venn diagram for identification of the intersection of the 65 differentially expressed IDEGs.


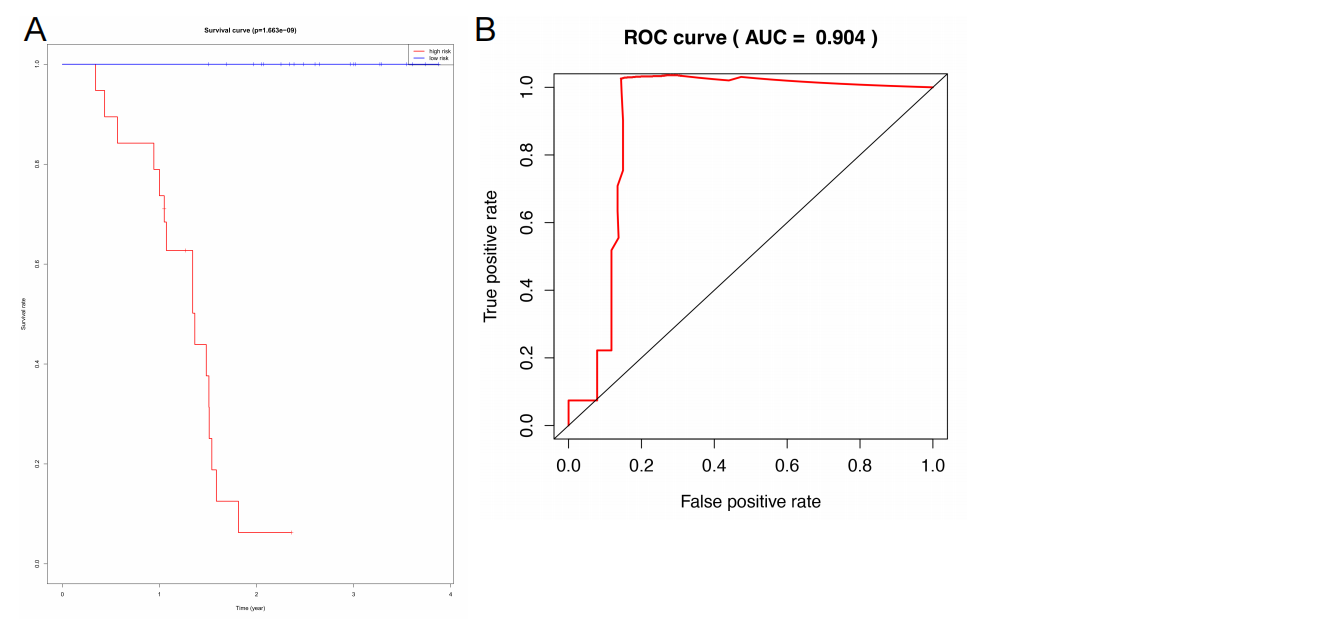


**Supplementary Figure 14.** Kaplan–Meier curve and ROC curve showing the predictive efficiency of the risk scores for the prognosis of ACC patients in the testing cohort.


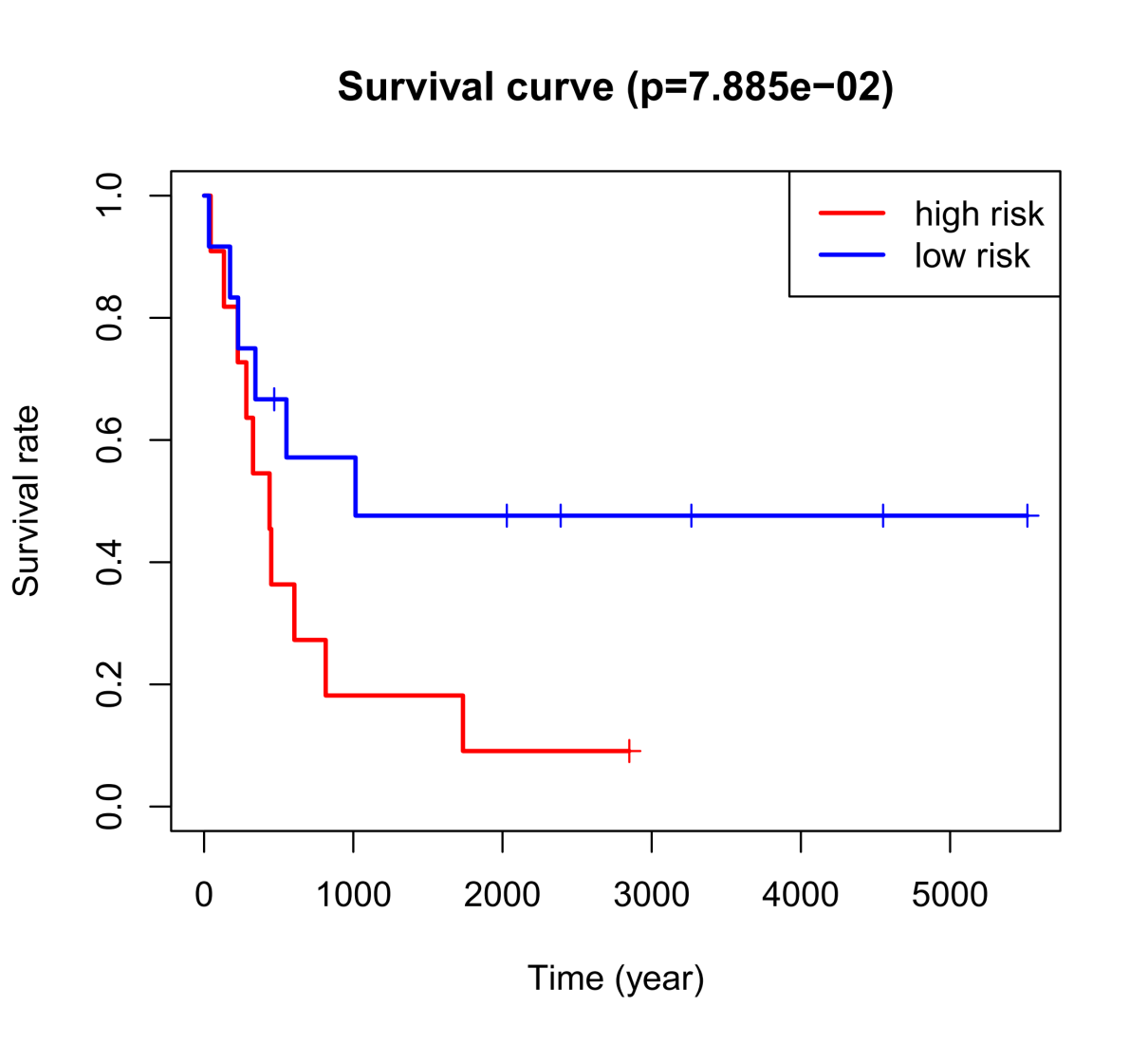


**Supplementary Figure 15.** Kaplan–Meier survival analysis of OS between high- and low- risk groups in external validation datasets.

# Supplementary Tables

**Supplementary Table 1**. Demographic characteristics of the tumor cohort used in this study

| Tumor  (n) | Noncancerous samples  (n) | Gender  (n) | | Stage  （n） | | | | T  (n) | | | | N  (n) | | | | M  (n) | |
| --- | --- | --- | --- | --- | --- | --- | --- | --- | --- | --- | --- | --- | --- | --- | --- | --- | --- |
|  |  | Male | Female | Stage I | Stage II | Stage III | Stage IV | T1 | T2 | T3 | T4 | N0 | N1 | N2 | N3 | M0 | M1 |
| BLCA  (408) | 19 | 305 | 109 | 4 | 130 | 142 | 136 | 5 | 119 | 196 | 60 | 239 | 46 | 77 | 8 | 202 | 11 |
| BRCA  (1093) | 112 | / | / | 181 | 619 | 242 | 18 | 277 | 629 | 139 | 35 | 514 | 358 | 116 | 76 | 902 | 20 |
| CESC  (304) | 3 | / | / | / | / | / | / | 140 | 72 | 21 | 10 | 134 | 61 | / | / | 116 | 11 |
| CHOL  (36) | 9 | 16 | 20 | 19 | 9 | 1 | 7 | 19 | 12 | 5 | / | 26 | 5 | / | / | 28 | 5 |
| COAD  (457) | 41 | / | / | 111 | 238 | 184 | 90 | 20 | 111 | 436 | 74 | / | / | / | / | / | / |
| DLBC  (48) | 0 | 22 | 26 | / | / | / | / | / | / | / | / | / | / | / | / | / | / |
| ESCA  (184) | 11 | 139 | 23 | 16 | 69 | 49 | 8 | 27 | 37 | 77 | 4 | 66 | 63 | 9 | 6 | 121 | 8 |
| GBM  (153) | 5 | 289 | 239 | / | / | / | / | / | / | / | / | / | / | / | / | / | / |
| HNSC  (520) | 44 | 368 | 134 | / | / | / | / | 33 | 144 | 131 | 179 | 239 | 80 | 154 | 7 | 472 | 5 |
| KICH  (66) | 25 | 39 | 26 | 20 | 25 | 14 | 6 | 20 | 25 | 18 | 2 | 39 | 3 | 2 | / | 34 | 2 |
| KIRC  (533) | 72 | 186 | 353 | 272 | 59 | 123 | 82 | 278 | 71 | 179 | 11 | 241 | 16 | / | / | 428 | 78 |
| KIRP  (290) | 32 | 212 | 77 | 172 | 22 | 51 | 15 | 193 | 33 | 59 | 2 | 49 | 24 | 4 | / | 95 | 9 |
| LAML  (173) | 0 | 83 | 68 | / | / | / | / | / | / | / | / | / | / | / | / | / | / |
| LGG  (516) | 0 | 289 | 239 | / | / | / | / | / | / | / | / | / | / | / | / | / | / |
| LIHC  (371) | 50 | 253 | 121 | 173 | 87 | 85 | 5 | 183 | 95 | 80 | 13 | 254 | 4 | / | / | 268 | 4 |
| LUAD  (515) | 59 | 286 | 249 | 294 | 123 | 84 | 26 | 175 | 289 | 49 | 19 | 348 | 95 | 74 | 2 | 361 | 25 |
| LUSC  (501) | 51 | 371 | 131 | 245 | 162 | 84 | 7 | 114 | 294 | 71 | 23 | 320 | 131 | 40 | 5 | 412 | 7 |
| MESO  (87) | 0 | 71 | 15 | 10 | 16 | 44 | 16 | 14 | 26 | 31 | 13 | 43 | 10 | 26 | 3 | 56 | 3 |
| OV  (303) | 0 | / | / | / | / | / | / | / | / | / | / | / | / | / | / | / | / |
| PAAD  (178) | 4 | 98 | 80 | 21 | 146 | 3 | 5 | 7 | 24 | 142 | 3 | 50 | 123 | / | / | 79 | 5 |
| PCPG  (179) | 3 | 81 | 102 | / | / | / | / | / | / | / | / | / | / | / | / | / | / |
| PRAD  (497) | 52 | / | / | / | / | / | / | 0 | 189 | 292 | 11 | 347 | 79 | / | / | 455 | 3 |
| READ  (166) | 10 | 91 | 75 | 30 | 51 | 51 | 24 | 9 | 28 | 113 | 14 | 84 | 45 | 33 | / | 126 | 23 |
| SARC  (259) | 0 | 119 | 144 | / | / | / | / | / | / | / | / | / | / | / | / | / | / |
| SKCM  (103) | 0 | 292 | 179 | 77 | 140 | 171 | 24 | 41 | 79 | 91 | 153 | 235 | 74 | 49 | 56 | 418 | 25 |
| STAD  (415) | 35 | 241 | 134 | 53 | 111 | 150 | 38 | 19 | 80 | 168 | 100 | 111 | 97 | 75 | 74 | 330 | 25 |
| TGCT  (150) | 0 | / | / | 106 | 12 | 14 | / | 80 | 52 | 6 | / | 51 | 11 | 2 | / | 4 | 51 |
| THCA  (501) | 59 | 139 | 371 | 286 | 52 | 113 | 57 | 143 | 167 | 175 | 23 | 229 | 231 | / | / | 286 | 9 |
| THYM  (120) | 0 | 62 | 57 | / | / | / | / | / | / | / | / | / | / | / | / | / | / |
| UCEC  (545) | 35 | / | / | / | / | / | / | / | / | / | / | / | / | / | / | / | / |
| UCS  (57) | 0 | / | / | / | / | / | / | / | / | / | / | / | / | / | / | / | / |
| UVM  (80) | 0 | 45 | 35 | / | 39 | 36 | 4 | 14 | 32 | 34 | 51 | / | / | / | / | 4 | 52 |

**Supplementary Table 2**. TCGA ACC patient characteristics.

| Clinical characteristics |  | Total (79) |
| --- | --- | --- |
| Age | <55 | 50 |
|  | ≥55 | 29 |
| Gender | Female | 31 |
|  | Male | 48 |
| Pathologic stage | Stage I | 9 |
|  | Stage II | 37 |
|  | Stage III | 16 |
|  | Stage IV | 15 |
| T | T1 | 9 |
|  | T2 | 42 |
|  | T3 | 8 |
|  | T4 | 18 |
| N | N0 | 68 |
|  | N1 | 9 |
|  | N2 | / |
|  | N3 | / |
| M | M0 | 62 |
|  | M1 | 15 |
| Mitotane therapy | No | 26 |
|  | Yes | 49 |
| Mitotic response rate | Mitotic rate>5/50 HPF absent | 29 |
|  | Mitotic rate>5/50 HPF present | 41 |
| Adrennal hormone history | Androgen | 6 |
|  | Androgen/cortisol | 10 |
|  | Cortisol | 8 |
|  | Estrogen | 2 |
|  | Mineralocorticoids | 2 |
| Therapy outcome | Complete remission/response | 46 |
|  | Partial remission/response | 1 |
|  | Progressive disease | 18 |
|  | Stable disease | 2 |
| Residual tumor | R0 | 53 |
|  | R1 | 5 |
|  | R2 | 9 |
|  | RX | 6 |

**Supplementary Table 3**.

| Description | SetSize | EnrichmentScore | NES | pvalue | p.adjust | qvalues | Rank | Leading_edge |
| --- | --- | --- | --- | --- | --- | --- | --- | --- |
| KEGG_CYTOKINE_CYTOKINE_RECEPTOR_INTERACTION | 250 | -0.33165178 | -1.662226821 | 0.001449275 | 0.031372549 | 0.024408848 | 5538 | tags=51%, list=28%, signal=37% |
| KEGG_LYSOSOME | 116 | -0.371821733 | -1.664202176 | 0.00155521 | 0.031372549 | 0.024408848 | 2969 | tags=29%, list=15%, signal=25% |
| KEGG_TOLL_LIKE_RECEPTOR_SIGNALING_PATHWAY | 97 | -0.37886444 | -1.660429169 | 0.001560062 | 0.031372549 | 0.024408848 | 5042 | tags=41%, list=26%, signal=31% |
| KEGG_HEMATOPOIETIC_CELL_LINEAGE | 83 | -0.413625802 | -1.762677661 | 0.0015625 | 0.031372549 | 0.024408848 | 6268 | tags=52%, list=32%, signal=36% |
| KEGG_OXIDATIVE_PHOSPHORYLATION | 117 | -0.407793259 | -1.825484034 | 0.001567398 | 0.031372549 | 0.024408848 | 2700 | tags=32%, list=14%, signal=28% |
| KEGG_GRAFT_VERSUS_HOST_DISEASE | 35 | -0.60011503 | -2.149917638 | 0.001628664 | 0.031372549 | 0.024408848 | 6137 | tags=69%, list=31%, signal=47% |
| KEGG_ALLOGRAFT_REJECTION | 33 | -0.617172228 | -2.167696792 | 0.001636661 | 0.031372549 | 0.024408848 | 6137 | tags=70%, list=31%, signal=48% |
| KEGG_AUTOIMMUNE_THYROID_DISEASE | 46 | -0.488507028 | -1.857794371 | 0.001652893 | 0.031372549 | 0.024408848 | 5756 | tags=70%, list=29%, signal=49% |
| KEGG_INTESTINAL_IMMUNE_NETWORK_FOR_IGA_PRODUCTION | 44 | -0.494678528 | -1.854323893 | 0.001672241 | 0.031372549 | 0.024408848 | 6137 | tags=55%, list=31%, signal=38% |
| KEGG_STEROID_BIOSYNTHESIS | 16 | 0.766246577 | 2.282919458 | 0.002352941 | 0.031372549 | 0.024408848 | 1936 | tags=69%, list=10%, signal=62% |
| KEGG_TERPENOID_BACKBONE_BIOSYNTHESIS | 15 | 0.730322721 | 2.124168161 | 0.002364066 | 0.031372549 | 0.024408848 | 3783 | tags=73%, list=19%, signal=59% |
| KEGG_PROPANOATE_METABOLISM | 31 | 0.620448729 | 2.287829681 | 0.002427184 | 0.031372549 | 0.024408848 | 3165 | tags=58%, list=16%, signal=49% |
| KEGG_VALINE_LEUCINE_AND_ISOLEUCINE_DEGRADATION | 43 | 0.56646885 | 2.206224619 | 0.00245098 | 0.031372549 | 0.024408848 | 3317 | tags=53%, list=17%, signal=45% |
| KEGG_PYRUVATE_METABOLISM | 40 | 0.5921999 | 2.269450574 | 0.0025 | 0.031372549 | 0.024408848 | 3110 | tags=50%, list=16%, signal=42% |
| KEGG_PEROXISOME | 77 | 0.418263799 | 1.860883177 | 0.002557545 | 0.031372549 | 0.024408848 | 3725 | tags=43%, list=19%, signal=35% |
| KEGG_PRIMARY_IMMUNODEFICIENCY | 35 | -0.493380357 | -1.767539689 | 0.003257329 | 0.037459283 | 0.029144523 | 3734 | tags=43%, list=19%, signal=35% |
| KEGG_CIRCADIAN_RHYTHM_MAMMAL | 11 | 0.759723385 | 2.062227909 | 0.004705882 | 0.046543002 | 0.036211946 | 517 | tags=36%, list=3%, signal=35% |
| KEGG_TYPE_I_DIABETES_MELLITUS | 39 | -0.495390167 | -1.824874013 | 0.004893964 | 0.046543002 | 0.036211946 | 6137 | tags=64%, list=31%, signal=44% |
| KEGG_FATTY_ACID_METABOLISM | 40 | 0.466939003 | 1.789421087 | 0.005 | 0.046543002 | 0.036211946 | 2904 | tags=40%, list=15%, signal=34% |
| KEGG_ASTHMA | 26 | -0.537536868 | -1.792135266 | 0.005059022 | 0.046543002 | 0.036211946 | 8027 | tags=69%, list=41%, signal=41% |
